# ROBITT: A tool for assessing the risk‐of‐bias in studies of temporal trends in ecology

**DOI:** 10.1111/2041-210X.13857

**Published:** 2022-04-06

**Authors:** Robin J. Boyd, Gary D. Powney, Fiona Burns, Alain Danet, François Duchenne, Matthew J. Grainger, Susan G. Jarvis, Gabrielle Martin, Erlend B. Nilsen, Emmanuelle Porcher, Gavin B. Stewart, Oliver J. Wilson, Oliver L. Pescott

**Affiliations:** ^1^ UK Centre for Ecology & Hydrology Wallingford UK; ^2^ RSPB Centre for Conservation Science Cambridge UK; ^3^ Centre d'Ecologie et des Sciences de la Conservation (CESCO), Muséum national d'Histoire naturelle, CNRS Sorbonne Université Paris France; ^4^ Swiss Federal Institute for Forest Snow and Landscape Research (WSL) Birmensdorf Switzerland; ^5^ Norwegian Institute for Nature Research (NINA) Trondheim Norway; ^6^ UK Centre for Ecology & Hydrology Lancaster Environment Centre Lancaster UK; ^7^ Laboratoire EDB Évolution & Diversité Biologique UMR 5174 Université de Toulouse, Université Toulouse 3 Paul Sabatier, UPS, CNRS, IRD Toulouse France; ^8^ Faculty of Biosciences and Aquaculture Nord University Steinkjer Norway; ^9^ Evidence Synthesis Lab, School of Natural and Environmental Science University of Newcastle Newcastle‐upon‐Tyne UK; ^10^ Plantlife Salisbury UK

**Keywords:** essential biodiversity variables, indicators, insect declines, risk‐of‐bias, species occurrence data, temporal trends, uncertainty

## Abstract

1. Aggregated species occurrence and abundance data from disparate sources are increasingly accessible to ecologists for the analysis of temporal trends in biodiversity. However, sampling biases relevant to any given research question are often poorly explored and infrequently reported; this can undermine statistical inference. In other disciplines, it is common for researchers to complete ‘risk‐of‐bias’ assessments to expose and document the potential for biases to undermine conclusions. The huge growth in available data, and recent controversies surrounding their use to infer temporal trends, indicate that similar assessments are urgently needed in ecology.
2. We introduce ROBITT, a structured tool for assessing the ‘Risk‐Of‐Bias In studies of Temporal Trends in ecology’. ROBITT has a similar format to its counterparts in other disciplines: it comprises signalling questions designed to elicit information on the potential for bias in key study domains. In answering these, users will define study inferential goal(s) and relevant statistical target populations. This information is used to assess potential sampling biases across domains relevant to the research question (e.g. geography, taxonomy, environment), and how these vary through time. If assessments indicate biases, then users must clearly describe them and/or explain what mitigating action will be taken.
3. Everything that users need to complete a ROBITT assessment is provided: the tool, a guidance document and a worked example. Following other disciplines, the tool and guidance document were developed through a consensus‐forming process across experts working in relevant areas of ecology and evidence synthesis.
4. We propose that researchers should be strongly encouraged to include a ROBITT assessment when publishing studies of biodiversity trends, especially when using aggregated data. This will help researchers to structure their thinking, clearly acknowledge potential sampling issues, highlight where expert consultation is required and provide an opportunity to describe data checks that might go unreported. ROBITT will also enable reviewers, editors and readers to establish how well research conclusions are supported given a dataset combined with some analytical approach. In turn, it should strengthen evidence‐based policy and practice, reduce differing interpretations of data and provide a clearer picture of the uncertainties associated with our understanding of reality.

## 1 INTRODUCTION

Species occupancy and abundance are fundamental state variables in ecology. Understanding the rates at which these variables are changing is required to monitor progress towards biodiversity targets and the effects of conservation interventions. Ultimately, this information comes from data documenting the detection of one or more individuals of some taxon; that is, species occurrence data, or, in some countries, ‘biological records’ (note that here we also use these terms to cover abundance data, as such information may be considered an occurrence attribute). Species occurrence data from disparate sources are often combined and analysed statistically to derive the measures of biodiversity over large taxonomic, spatial and temporal extents (e.g. Gregory et al., 2005). Indeed, this is the premise of species population ‘essential biodiversity variables’ (Jetz et al., 2019; Kissling et al., 2018; Pereira et al., 2013). The temporal component of these data products may be averaged over spatial and taxonomic domains to produce indicators (GEO BON, 2015); these have become a key source of information on ecological change for policymakers (Navarro et al., 2017). Frequently then, evidence of temporal trends in biodiversity is derived through the statistical analysis of species occurrence data.

Species occurrence data vary widely in terms of why and how they were recorded, and the information that they provide. Presence‐only data document the sighting of some species, with information on where and when the sighting occurred. These data are derived from a variety of sources, including natural history collections in museums and herbaria, surveys by professional biologists and various types of data collected by volunteer naturalists (Collen et al., 2013). Presence–absence data provide additional information on sampling events which did not yield a detection of the focal taxon. These data are most likely to be collected through structured monitoring schemes using specific protocols (but see Sullivan et al., 2014). Abundance data can provide more information still: they document the number (or other quantity) of individuals detected. All of these data can be used to provide information on trends in biodiversity.

In recent years, species occurrence data have increased in volume and accessibility. This can be ascribed to several initiatives: the digitisation of historic biological records (Page et al., 2015); the proliferation and growth of citizen science monitoring initiatives (Spear et al., 2017); the launch of online data aggregators such as GBIF and similar regional portals (Nelson & Ellis, 2019); and the compilation of more specialist databases focused on particular types of ecological community (Dengler et al., 2011), monitoring data (Dornelas et al., 2018) or other evidence types (Hudson et al., 2017). Thanks to these initiatives, it is now straightforward for ecologists to access large quantities of data, and to use them for research. However, data quantity does not necessarily equal quality of scientific insight, and there have been important questions raised concerning the suitability of some biodiversity data for drawing reliable inferences about change over time (e.g. Ball‐Damerow et al., 2019; Cardinale et al., 2018; Pescott et al., 2019).

To appreciate the potential challenges associated with the analysis of heterogeneous data, it is useful to define some key statistical concepts (see Box 1 in Supporting Information 2 for a glossary of relevant terms). While there are many possible definitions of statistics (Barnett, 1982), one typical conception is that of reasoning under uncertainty and inherent variability, with classical texts (e.g. Lehmann, 1959) focusing on the use of observed data to make inferences concerning unobserved distributions. For example, monitoring‐type investigations can be appreciated as a sample‐based approach to understanding features of some broader environment; likewise, smaller scale experiments are normally conducted with generalisation in mind. In both these cases, it is rarely feasible to census an entire population of interest: researchers use samples. This leads to questions concerning the validity of inferences. One assessment of a study's validity is to ask whether these inferences are well‐supported by the data in hand (internal validity). For sample‐based results to be generalisable, however, they must also be true of the wider population of interest (external validity). A study's external validity is likely to be undermined if samples are not representative of the population with respect to important features for the desired inferences (Meng, 2018); this is often known as ‘sampling bias’ or, sometimes, ‘selection bias’.

To obtain a representative sample, researchers would ideally select individual units randomly from the population (probability sampling). However, this is often impractical, in which case researchers might make use of non‐probability samples, such as those found in aggregated biodiversity databases; these are samples that were not necessarily collected to be representative of a clearly defined population. Small samples may also be unrepresentative of important features by chance, even if they are probability samples. Before researchers can understand a sample's representativeness, they must first define their research question and statistical target population.

In studies of biodiversity trends, researchers tend to define their statistical populations along the axes of space, time and taxonomy (e.g. Dennis et al., 2019; Outhwaite et al., 2019; Powney et al., 2019; van Strien et al., 2019). For example, one might be interested in trends in bird distributions in North America over the period 1950 to the present day. It is also worth noting that, although they may not always be defined explicitly, other axes may be important for inference. For example, researchers may be more interested in whether samples represent all areas of some multidimensional environmental space (e.g. as defined by a set of climatic variables), rather than just being considered representative of geographic space. Likewise, for some purposes, representative coverage of species' traits may be desired along with, or instead of, even phylogenetic coverage. To be representative of such populations, data should be representative of all axes. To illustrate this point using the above example, data would need to be sampled as close to randomly as possible across North America, across all relevant bird species, and evenly between 1950 and the present day. Otherwise, it is possible that the data will be unrepresentative of the populations of interest. For example, particular geographical areas may be over‐ or undersampled at particular times, leading to a confounding of time and space, and, ultimately, conclusions that bear little resemblance to the true state of nature.

There are many situations in which occurrence data are unlikely to be representative of the statistical populations implied in studies of biodiversity trends. Data collected opportunistically are highly likely to be non‐random along the key axes of space, time and taxonomy (or other important dimensions). Volunteer naturalists, for example, tend to preferentially sample accessible and attractive locations, and interesting species (Barends et al., 2020; Prendergast et al., 1993). Structured data, collected according to some sampling design, may well be representative of some set of domains; however, when multiple datasets, with different aims, extents and protocols, are aggregated (e.g. as on GBIF), then the target population to which these data pertain becomes unclear. To illustrate this point, imagine several datasets, each derived from structured monitoring of some taxon in some spatial unit at regular time intervals. These data might be very informative about change in those units (but see Gonzalez et al., 2016), but there is no reason to suppose that they can be combined and used to draw robust inferences about some wider geographic domain, unless the samples happen to resemble a probability sample of the broader population(s) of interest (Cardinale et al., 2018). The problem of a mismatch between sample and population could be reduced or avoided if researchers first assessed their data to inform readers of their choice of population and the scope of their inferences.

The frequent mismatch between sample and statistical target population in studies of biodiversity trends has not gone unnoticed; indeed, it is a common subject for critical comments on studies in the literature. For example, Sánchez‐Bayo and Wyckhuys (2019) and van Klink et al. (2020) were criticised for extrapolating their claims of insect declines beyond the taxonomic and geographical limits of their data (Desquilbet et al., 2020; Jähnig et al., 2021; Saunders et al., 2020; Simmons et al., 2019). Vellend et al. (2013) and Dornelas et al. (2014) were criticised for concluding that local species richness is not in decline globally from meta‐analyses of studies that were geographically biased in relation to human disturbance and species richness itself (Cardinale et al., 2018; Gonzalez et al., 2016). Crossley et al. (2020) and van Klink et al. (2020), on the other hand, were taxonomically selective when reporting their conclusions: both sets of authors included non‐insect groups in their analyses, but restricted their conclusions (and paper titles) to insects (Desquilbet et al., 2020, 2021). Other studies of insect trends have been criticised with regard to whether particular modelling approaches have appropriately dealt with temporal biases in the data. For example, both Lister and Garcia (2018) and Soroye et al. (2020) have been criticised in this regard (Anon., 2020; Guzman et al., 2021; Willig et al., 2019). This brief overview of some recent disagreements highlights a fundamental problem: potential biases are rarely communicated to the reader in sufficient detail; instead, they are often addressed with a passing comment, if at all.

In other disciplines, strategies have developed to assist researchers in avoiding potentially inappropriate inferential claims. In medicine and related areas, inclusion of a study in a systematic review often requires that the original publication is subject to a ‘risk‐of‐bias’ (RoB) assessment. Several tools have been developed to conduct RoB assessments, each focusing on a particular type of study and data (see Supporting Information 5). While many of these tools were designed for use in systematic reviews, others were designed for use at the primary research stage or both (Supporting Information 5). Regardless, the function of these tools is essentially the same: to clearly expose threats to the validity of a study's conclusions arising from potential biases in the underlying data. RoB tools in medicine have been described as reflecting a ‘shift in focus from methodological quality to risk of bias’ (Sterne et al., 2016)—a shift that has yet to take place in ecology, despite efforts to provide structured approaches to documenting methodological choices in some areas (e.g. Grimm et al., 2010). It is easy to appreciate why this shift was needed in medicine: one would not want to approve some pharmaceutical product which had only been demonstrated to be safe in some population subset, for example. We argue that the increasing policy relevance of inferences about trends in biodiversity necessitates a similar transition in ecology.

In this paper we introduce ROBITT, a tool for assessing the ‘Risk‐Of‐Bias In studies of Temporal Trends in ecology’. The tool has a similar format to its counterparts in other fields: it comprises a number of ‘signalling’ questions (Sterne et al., 2016) designed to elicit information on the potential for bias in a study. Users are first asked to define the statistical target population about which they intend to make inferences, and then to assess whether their data are likely to be representative of this population in the geographic, temporal, environmental and taxonomic domains as relevant (the latter defined broadly as covering any organismal space that might be important for inference). If the data are found to be potentially biased, then the user is asked to explain how they will mitigate those biases, or how they will be clearly and appropriately communicated. Below we describe the development of the tool, provide an overview, describe its sections and refer the reader to the Supporting Information for the tool itself, a guidance document and a worked example. Finally, we discuss the potential value of ROBITT for ecology and propose its inclusion as Supporting Information for all studies of biodiversity trends based on species occurrence data—particularly where those data are obtained from aggregated databases.

## 2 ROBITT TOOL

### 2.1 Development

ROBITT was developed through a consensus‐forming process involving experts across relevant areas of ecology and evidence synthesis (the authors; see Supporting Information 3 for details).

### 2.2 Overview

**FIGURE 1:**
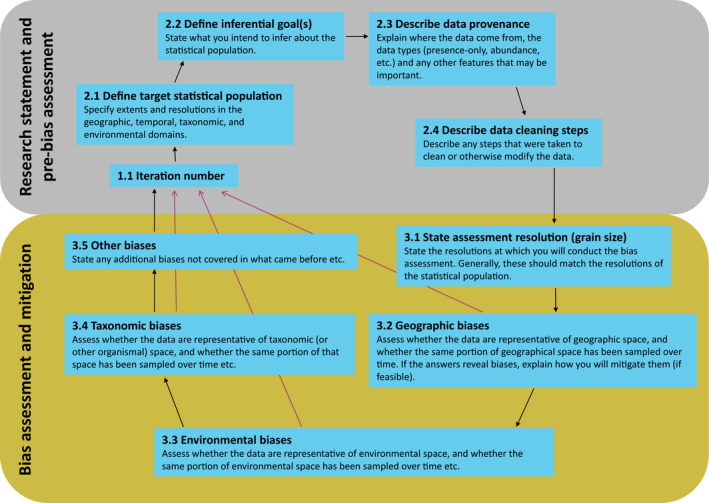
A conceptual overview of ROBITT with brief details about what is required at each stage. Black arrows indicate the order in which users should proceed through a ROBITT assessment. Purple arrows indicate that completing a ROBITT form can be an iterative process: if the data are found to be unrepresentative of any domain, then they may be necessary to return to step 1.1 and redefine the extent and/or resolution of the statistical population accordingly

ROBITT comprises 17 questions designed to elicit information on a study's potential for bias. The user may answer the questions using text and/or figures. The first section, the ‘research statement and pre‐bias assessment’, comprises four questions concerning the scope of the research and related issues; the remainder constitutes the bias assessment itself. See Figure 1 for an overview of the tool. The ROBITT tool and supporting guidance document can be found in Supporting Informations 1 and 2. The guidance follows the PRISMA model (Page et al., 2021): that is, an explanation of the rationale for each question is given, followed by a summary of the expected response. Worked examples of ROBITT are provided in Supporting Information 4.

### 2.3 Tool sections

#### 2.3.1 Research statement and pre‐bias assessment

The purpose of this section is to assemble the information needed to assess a study's RoB. The first step is to define the target population about which inferences are desired. This must include a specification of the extents of any relevant domains (e.g. geographic, temporal, taxonomic, environmental). It must also include a statement of the resolutions at which analyses will be conducted (e.g. 1 km grid cells, annual increments etc.). This is important because the scale at which a research question is formulated can influence data availability and the nature of, and potential for, biases (e.g. Pescott et al., 2019). The next step is to state the inferential goal; for example, ‘to estimate temporal trends in species' occupancy’. In the remainder of this section, the user must document data provenances and explain and justify any steps that were taken to modify or clean data.

#### 2.3.2 Bias assessment

The main section of ROBITT is the bias assessment. This begins with a specification of the geographic, temporal and taxonomic resolutions (grain sizes) at which the assessment will be conducted. Generally, these should match the resolutions at which inferences are desired (as specified in the research statement section). It would likely be inappropriate, for example, to assess data in decadal time periods and 100 km grid cells, and then conclude that they were unbiased for making yearly inferences at the 1 km resolution. We note that there may be limited exceptions to this: for example, it is not possible to assess sampling biases at the species level using presence‐only data because these say nothing about sampling effort where the focal species was not observed.

The next three subsections denote our three main domains of potential bias: geographic, environmental and taxonomic (or other organismal axis, such as functional group). Temporal biases are dealt with each of these three sections (see below). In each subsection, the user must answer three questions: the first two are designed to reveal potential biases relative to the research question (i.e. the inferential goal). The first question asks whether the data are representative of that domain; that is to say, do the data cover the whole domain evenly (ideally randomly)? The second question asks whether the same portion of the focal domain has been sampled over time; that is, is there any indication of temporal changes in coverage? The answers to this second question are crucial for assessing the suitability of the data for estimating temporal trends. To illustrate this, imagine that species data are collected from one location in one time period, and then from another in the next. Using these data to estimate changes in species' distributions or abundances between time periods will likely be problematic, because shifts in space are confounded with shifts in time. In one sense, the distinction between the first and second questions can be considered equivalent to the distinction between external and internal validity: a study might have low external validity if it is not representative of some domain overall; however, for a subset of that domain (e.g. a well‐sampled portion of geographic space), the data might be very informative about change (i.e. high internal validity). The answers to these first two questions in each domain have important implications for how one answers the third.

The third question in each domain subsection asks the user to state how they will mitigate potential biases indicated by the preceding two questions. There are several ways in which one might go about mitigating biases, which we review in the Discussion. There will be cases in which it is unnecessary to mitigate for a lack of coverage or inconsistent sampling over time because these are not relevant to the inferential goal. For example, even coverage in environmental space may be inappropriate if environmental change is expected over time for the geographic extent of the analysis. Users are not required to explain poor coverage in any domain if it is irrelevant to their inferences. There could also be situations in which a bias is deemed relevant but mitigation is not feasible. In this case, the resultant trends should be appropriately and clearly caveated.

The final subsection is ‘Other potential biases’. This is different to the previous three in that it does not relate to a single domain; rather, it provides an opportunity for the user to consider additional biases that might affect their research. The first question asks whether there are any temporal biases that do not relate to the ecological states of interest. Often these biases will relate to observation error or the estimation of some parameter in a model related to this. For example, site occupancy models are sometimes used to estimate trends in species' occupancies (Kéry & Royle, 2016). These models normally require data from replicate visits to sites within short spaces of time to estimate detection probabilities (thus correcting for imperfect detection). Where these models are used, analysts should consider whether there is variation in the quantity and type of repeat visits that could result in biased estimates of these parameters (Royle, 2006).

The second question in the ‘other biases’ section asks the user to consider whether there are any other biases not covered by the preceding questions. Examples include biases relating to phenology, such as a mismatch between sampling dates and a species' flight period; temporal baselines; and changes in the portion of one domain that has been sampled over some other domain, such as geographic variation in taxonomic coverage. Like earlier sections, the final question asks users to explain how they plan to mitigate biases revealed in their answers to the two preceding questions. See the guidance document in Supporting Information 2 for details on the expected content of responses to the ROBITT questions and other background information.

#### 2.3.3 Completing the assessment

While the assessment questions require individual answers, it may be that researchers prefer to provide responses in the main text of a report. As a point of comparison, PRISMA (Page et al., 2021) provides a checklist format that allows researchers to direct the reader to the answer to any given question. This could also be the case here; for example, paper subheadings could be provided in response to a question, provided the text referenced was a complete answer to it.

**FIGURE 2:**
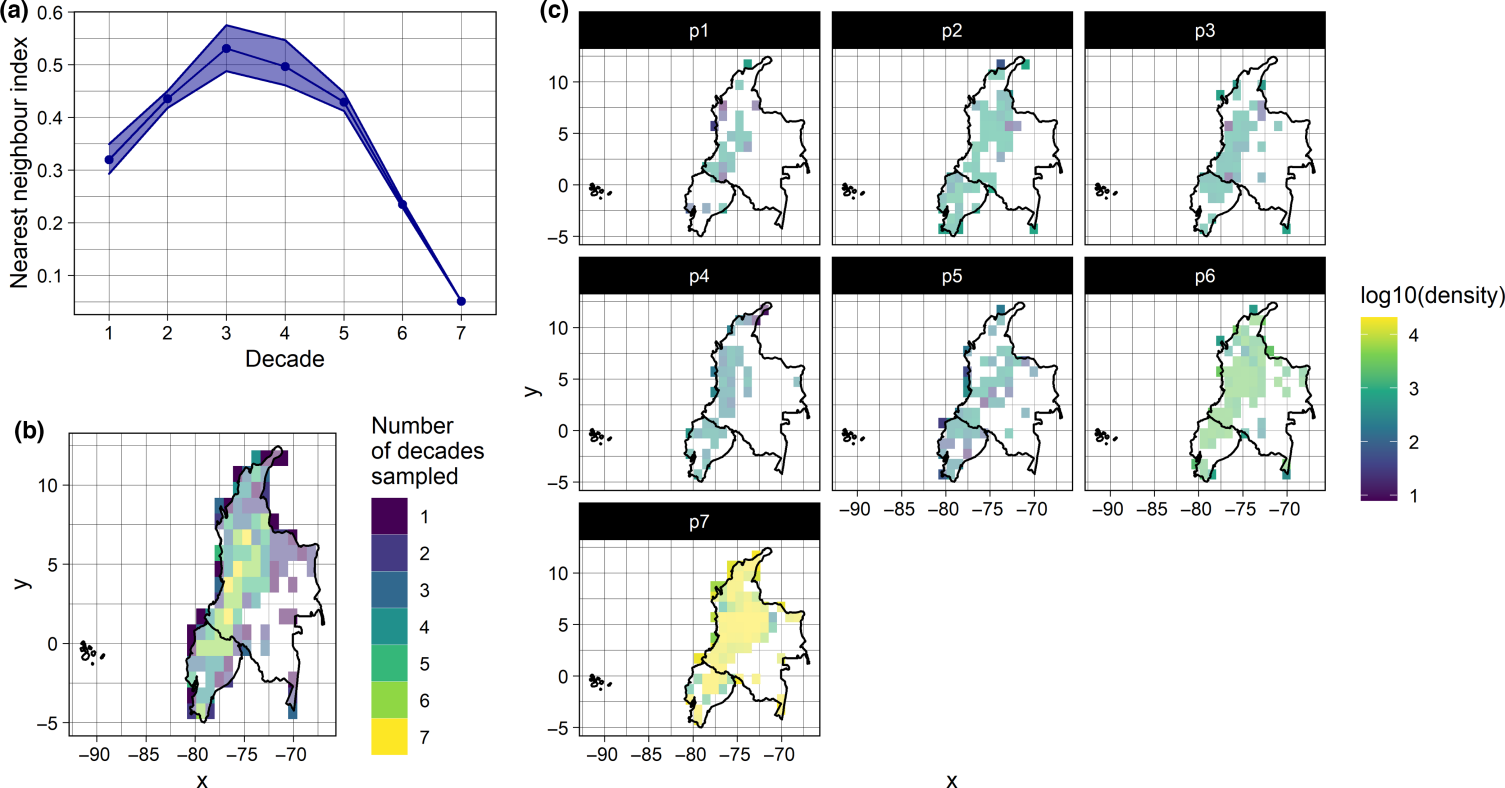
Three ‘heuristics’ indicating the potential for geographic biases in data on hummingbird occurrences collected in Ecuador and Colombia from 1950 to 2019. These data were downloaded from GBIF (see Supporting Information 3 for full details of the provenance of these data). In these examples, the data are assessed in seven decadal time periods (p1 = 1950–1959, p2 = 1960–1969, etc.) and in 1° grid cells. Panel (a) shows the nearest neighbour index for each decade; values further from 1 indicate a greater departure from a simulated random distribution. The shaded band denotes uncertainty derived by bootstrapping. Panel (b) is a map showing the number of decades in which records are available for each grid cell. This is a simple measure of how the spatial distribution of sampling has changed over time. Panel (c) shows the density of records in each grid cell for each decade on a log10 scale. See Boyd et al. (2021) for further details

Users may go about answering the questions in the bias assessment section in the best ways they see fit. However, we have found the use of ‘heuristics’ that indicate the potential for bias to be of value. We use the term ‘heuristic’ to acknowledge that it is generally not possible to determine the exact extent of bias without a probability sample for comparison. Many heuristics have been used to screen biodiversity data for biases in the literature; we briefly review these in Table S1 in Supporting Information 2. The most common example is a map of the density of records across geographic space; such maps could provide evidence of geographic representativeness (or lack thereof). Taking this further, one could produce several maps, each pertaining to some time period; these could be used to assess temporal variation in geographic coverage. To obtain a more formal, quasi‐statistical measure of geographic representativeness, one could compare the nearest neighbour distances of their data to those of a simulated random distribution (Clark & Evans, 1954). This gives an index indicating the extent to which the data depart from a random distribution geographically. In Figure 2, we present three example heuristics that could be used to screen data for geographic biases. In these examples, the heuristics are applied to hummingbird (Trochilidae) records collected between 1950 and 2019 in Ecuador and Colombia. While heuristics of this type will be useful, it is important to remember that a ROBITT assessment is not intended to be a contextless set of numbers or figures: bias can strictly only be defined in relation to some inferential goal. The central point of ROBITT is that assessments of bias are clearly linked to a research question, and assessed in the context of this and any analytical tools being used to answer that question.

In some cases, completing a ROBITT assessment will be an iterative process. For example, researchers might complete a first iteration of the tool and find that data coverage is not sufficient in portions of their geographic domain of interest. In this case, they might decide to redefine this domain to exclude poorly sampled regions; this would mean completing a second iteration of ROBITT using an appropriate subset (Figure S1, see Supporting Information 4). Where a ROBITT assessment is iterative, the user should clearly version control (i.e. track and record changes over time) their documents and provide this history as supporting information to their work.

## 3 DISCUSSION

Sampling biases have long been recognised as a challenge for inference in ecology (e.g. Peters, 1991); however, unlike in other disciplines, no formal tools for assessing these have been produced. We have designed and introduced ROBITT, a tool for assessing the potential ‘Risk‐Of‐Bias in studies of Temporal Trends in ecology’. The tool comprises a number of questions, each designed to clearly elicit the potential for bias in the study under assessment. In answering these, users will define their research question and target population across relevant domains, and then assess the degree to which their data are likely to be representative of these. We propose that researchers be strongly encouraged to include a ROBITT assessment as supporting information when publishing studies of temporal trends in biodiversity, especially when using aggregated data. We expect that this will support scientists in writing clear method sections; strengthen evidence‐based policy and practice; help resolve scientific controversies around biodiversity trends; assist editors, reviewers and readers; and, ultimately, highlight the uncertainty associated with our understanding of ecological reality. Accumulated over studies, ROBITT assessments will also highlight where data are required to address pressing questions concerning biodiversity change.

We hope that the completion of ROBITT will become a standard requirement where researchers estimate trends from aggregated species occurrence data. The tools listed in Supporting Information 5 have set similar precedents in other disciplines; many are endorsed by journals and uptake is generally high. While some reporting tools for various subdisciplines of ecology already exist, they do not focus on RoB. These include the ODD (Grimm et al., 2006, 2010) and TRACE (Schmolke et al., 2010) protocols for describing and documenting individual‐based models, and the ODMAP (Zurell et al., 2020) protocol for documenting the use of species distribution models. In medicine, some reporting tools have evolved from a general focus on methodology to a more specific, and arguably more in‐depth, focus on the impacts of bias on inference (Sterne et al., 2016). There is no doubt a place for both in ecology (indeed, some tools in medicine combine these aspects, e.g. Page et al., 2021); however, we agree with Sterne et al. (2016) that in‐depth, qualitative, assessments of RoB across relevant domains are more useful and revealing than simply checking methodological items off a list.

We suggest that researchers will get the greatest benefit from our tool if they use it to structure their research. ROBITT contains questions that researchers should be asking themselves already; indeed, it provides an opportunity to demonstrate the large amount of work that goes into studies of temporal trends in biodiversity, but which may go unreported. An interesting possibility is that ROBITT assessments could be supplied as part of the pre‐registration process, which is becoming increasingly common in ecology (e.g. https://besjournals.onlinelibrary.wiley.com/hub/journal/26888319/registered‐reports‐author‐guidelines). If, on the other hand, a ROBITT form is completed just before the submission of an article for publication, then it may reveal problems that could have been dealt with earlier. Completing the form during the research process has the potential to save researchers' time, by providing a framework for structuring thought and decision‐making.

Much of the RoB literature in other disciplines has focused on the effects of interventions (see Supporting Information 5). In this type of research, the questions asked are causal because the desired inference concerns whether some action results in some outcome. This has also been the standard focus of evidence‐based conservation (e.g. Lortie et al., 2015). ROBITT, on the other hand, is primarily focused on descriptive inference of the type that is often used for ecological indicators (e.g. Gregory et al., 2005) or the EBV literature (e.g. Jetz et al., 2019). However, this distinction is not absolute, and there are many examples of ecological studies that use aggregated species occurrence data in attempts to reach causal conclusions. For example, Woodcock et al. (2016) divided wild bee data for Britain into two subsets based on insecticide use, assessing trends in occupancy for taxa in each subset. While this type of assessment is correlative, there is often a causal motivation (e.g. the title of Woodcock et al., 2016 implies causality). While the ROBITT tool has not been explicitly designed to deal with these situations, we suggest that it will still be useful when attempting to make causal inferences from observational data. In the example of Woodcock et al. (2016), the domain representativeness of the data in the two subsets could have been assessed separately to investigate the potential for confounding; additionally, the full dataset could have been assessed for its external validity.

One key issue with RoB assessments is that, while it might be easy to define a target population, in some cases it will not be straightforward to determine whether any given sample is representative of that population. For example, a researcher might define their population as wild bees in Chile in the 2010s. Mapping the data might reveal that available data are not randomly distributed across the country, but does this reflect the true distribution of wild bees in Chile, or does it reflect non‐random sampling? The user might also want to establish whether they have data for all known species of wild bee in Chile: how do they know whether this is the case? The answers to these questions will vary.

While it will not always be easy to establish whether a sample is representative of a population, we propose some simple criteria. First, subject‐matter experts should be consulted; experts may be able to separate sampling biases from biological phenomena. For example, an expert might know, or suspect, that a species or taxon group occupies areas where it has not been recorded; this is likely to be a strong indication of sampling bias. Second, it might be possible to supplement expert advice with published information. Regional or national floras etc. may list (undigitised) specimens, or provide information on regional occurrences at some coarse spatio‐temporal level. Third, when using presence‐only data for a reasonably large number of species in the same group (e.g. bees, birds), it may be acceptable to assume that the combined distribution of records for all species approximates the sampling distribution (Dudík et al., 2005; Phillips et al., 2009). In this case, the combined data would ideally be randomly distributed across the geographical domain. Fourth, presence/absence and abundance data may be a direct reflection of the distribution of sampling (i.e. a species might not be detected but a record is still made of the event), therefore such data may provide reliable information on the distribution of sampling in space and time. If the basis of sampling is known (e.g. random, systematic‐random etc.), then data may be representative, at least within the bounds of the original survey. However, even here, such a sample may still be unrepresentative of an analyst's target population if that population pertains to a different spatio‐temporal‐taxonomic domain to the survey. We can see very few scenarios where it will not be possible to at least approximate the degree to which a dataset is representative of a given population using all the knowledge that could be brought to bear. Indeed, this is the rationale behind qualitative RoB tools based on expert assessments (Supporting Information 5).

If analysts cannot reach an informed conclusion with regard to the likely representativeness of a sample, then broader inference is not likely to be meaningful; simple descriptive statistics could be used instead, and this limitation acknowledged, with paper titles, abstracts etc. all reflecting this. This may seem a negative conclusion for an analyst to reach, but we argue that this is likely to be the most honest, and scientific, endpoint for a dataset whose representativeness cannot be clearly assessed.

Four of the questions in ROBITT provide researchers with an opportunity to consider whether and how they can mitigate biases revealed elsewhere in the tool. It is not possible to review here all possible measures that could be taken by researchers; a full treatment of adjustments and models for dealing with bias would have to cover many topics within statistics and ecological data. However, we note three general approaches. The first is to modify the data in some way (e.g. thinning; Inman et al., 2021). The second is to model the biases; typically, this will involve incorporation of variables thought to capture the biasing mechanism in some form of regression analysis (e.g. van Strien et al., 2019), although other approaches are possible (Ahmad Suhaimi et al., 2021). Third, we suspect that in many cases ROBITT will reveal the need to restrict the extent of researchers' inferences. This might include redefining the spatial extent of an analysis to reflect the fact that data are scarcely available in some portion of geographic space, or coarsening the temporal resolution to ‘smooth over’ temporal biases in geographic or taxonomic coverage (Pescott et al., 2019). Any modifications to the extents of the statistical population should be reflected in paper titles, abstracts, etc. We note that it will often be prudent for researchers to assess the sensitivity of their conclusions to the choice of bias mitigation strategy: some statistical ‘fixes’ can make aspects of inference worse (Gelman, 2007; Lele, 2010). Nevertheless, we suspect that by using these general bias mitigation strategies, researchers will usually be able to proceed with their analyses, even if those analyses relate to more limited statistical populations than initially envisioned.

The problem of inference from biased samples is difficult, and quick fixes do not exist. ROBITT represents a first attempt to encourage more thoughtful assessment of the potential for bias to undermine the robust estimation of temporal trends in ecology. We intend to update the tool over time and welcome feedback from users.

## CONFLICT OF INTEREST

The authors have no conflict of interest to disclose.

## AUTHORS' CONTRIBUTIONS

R.J.B. and O.L.P. conceived the idea and led the writing of the manuscript; R.J.B., O.L.P. and G.D.P. designed the methodology; all authors provided critical feedback on the methodology and manuscript, and attended two workshops at which ROBITT was developed.

### PEER REVIEW

The peer review history for this article is available at https://publons.com/publon/10.1111/2041‐210X.13857.

## Supporting information


**Appendix S1** Supplementary Material 1

**Appendix S2** Supplementary Material 2

**Appendix S3** Supplementary Material 3

**Appendix S4** Supplementary Material 4

**Table S1** Supplementary Material 5

## Data Availability

There are no data or code associated with this manuscript.
