# Supplementary material for: ROBITT: A tool for assessing the risk‐of‐bias in studies of temporal trends in ecology
Source: Methods Ecol Evol. 2022 Apr 6;13(7):1497–507. doi: 10.1111/2041-210X.13857 (PMC9541136; doi:10.1111/2041-210X.13857)
Supplement: Supplementary file 1 — Appendix S1 Supplementary Material 1 [file MEE3-13-1497-s004.docx]

[This page can be deleted.]

# ROBITT (“Risk Of Bias In studies of Temporal Trends in ecology”) tool

Please ensure that you understand the premise of this document as outlined in the following paper and the associated guidance document:

Boyd, R.J., Powney, G.D., Burns, F., Danet, A., Duchenne, F., Grainger, M., Jarvis, S.G., Martin, G., Nilsen, E.B., Porcher, E., Stewart, G.B., Wilson, O.J., Pescott, O.L. 2022. ROBITT: a tool for assessing the risk of bias in studies of temporal trends in ecology. *Methods in Ecology and Evolution*

To complete this form, please delete all instances of <insert text> and replace with your own text and/or figures.

# Contents

[ROBITT (“Risk Of Bias In studies of Temporal Trends in ecology”) tool](#_gjdgxs) 1

[Contents](#_30j0zll) 1

[Iteration](#_1fob9te) 2

Research statement and pre-bias assessments 2

[Statistical population of interest](#_3znysh7) 2

[Inferential goals](#_2et92p0) 2

[Data provenance](#_tyjcwt) 2

[Data processing](#_3dy6vkm) 2

[Bias assessment and mitigation](#_1t3h5sf) **2**

[Assessment resolution](#_4d34og8) 2

[Geographic domain](#_2s8eyo1) 3

[Environmental domain](#_17dp8vu) 3

[Taxonomic domain (or other organismal domain, e.g., phylogenetic, trait space etc.)](#_3rdcrjn) 3

[Other potential biases](#_26in1rg) 3

[Supporting references](#_35nkun2) 4

# Iteration

**1.1 ROBITT iteration number**

| **Iteration** | **Comments** |
| --- | --- |
|  |  |

# Research statement and pre-bias assessments

## Statistical population of interest

**2.1 Define the statistical target population about which you intend to make inferences.**

| **Domain** | **Extent** | **Resolution** |
| --- | --- | --- |
| Geographic | <insert text> | <insert text> |
| Temporal | <insert text> | <insert text> |
| Taxonomic (or other relevant organismal domain such as functional group etc.) | <insert text> | <insert text> |
| Environmental | <insert text> | <insert text> |

## Inferential goals

**2.2 What are your inferential goals?**

<insert text>

## Data provenance

**2.3 From where were your data acquired (please provide citations, including a DOI, wherever possible)? What are their key features in respect of the inferential aims of your study (see the guidance document for examples)?**

<insert text>

## Data processing

**2.4 Provide details of, and the justification for, all of the steps that you have taken to clean the data described above prior to analyses.**

<insert text>

# Bias assessment and mitigation

## Assessment resolutions

**3.1 At what geographic, temporal and taxonomic resolutions (i.e. scales or grain sizes) will you conduct your bias assessment?**

<insert text>

## Geographic domain

**3.2 Are the data sampled from a representative portion of geographical space in the domain of interest?**

<insert text>

**3.3 Are your data sampled from the same portions of geographic space across time periods?**

<insert text>

**3.4 If the answers to the above questions revealed any potential geographic biases, or temporal variation in geographic coverage, please explain, in detail, how you plan to mitigate them.**

<insert text>

## Environmental domain

**3.5 Are your data sampled from a representative portion of environmental space in the domain of interest?**

<insert text>

**3.6 Are your data sampled from the same portion of environmental space across time periods?**

<insert text>

**3.7 If the answers to the above questions revealed any potential environmental biases, or temporal variation in environmental coverage, please explain, in detail, how you plan to mitigate them.**

<insert text>

## Taxonomic domain (or other organismal domain, e.g., phylogenetic, trait space etc.)

**3.8 Is the sampled portion of the taxonomic (or phylogenetic, trait or other space if more relevant) space representative of the taxonomic (or other) domain of interest?**

<insert text>

**3.9 Do your data pertain to the same taxa/taxonomic domain across time periods?**

<insert text>

**3.10 If the answers to the above questions revealed any potential taxonomic biases, or temporal variation in taxonomic coverage, please explain, in detail, how you plan to mitigate them.**

<insert text>

## Other potential biases

**3.11 Are there other potential temporal biases in your data that relate to variables other than ecological states?**

<insert text>

**3.12 Are you aware of any other potential biases not covered by the above questions that might cause problems for your inferences?**

<insert text>

**3.13 If questions 3.11 or 3.12 revealed any important potential biases, please explain how you will mitigate them.**

<insert text>

# Supporting references

<insert text>
