# Supplementary material for: ROBITT: A tool for assessing the risk‐of‐bias in studies of temporal trends in ecology
Source: Methods Ecol Evol. 2022 Apr 6;13(7):1497–507. doi: 10.1111/2041-210X.13857 (PMC9541136; doi:10.1111/2041-210X.13857)
Supplement: Supplementary file 2 — Appendix S2 Supplementary Material 2 [file MEE3-13-1497-s002.docx]

ROBITT (“Risk Of Bias In studies of Temporal Trends in ecology”): Guidance document

Boyd, R.J., Powney, G.D., Burns, F., Danet, A., Duchenne, F., Grainger, M., Jarvis, S.G., Martin, G., Nilsen, E.B., Porcher, E., Stewart, G.B., Wilson, O.J., Pescott, O.L. 2022. ROBITT: a tool for assessing the risk of bias in studies of temporal trends in ecology. *Methods in Ecology and Evolution*

General guidance and background

This document is the guidance for the “Risk Of Bias In studies of Temporal Trends in ecology” (ROBITT) tool. The tool itself is a series of questions designed to prompt researchers to fully and transparently evaluate the potential risk of bias in studies that use data on species’ occurrences and/or abundances, or related data types, to estimate temporal trends in biodiversity. The development of the tool has been primarily motivated by: (i) the growth in primary biodiversity data, and its availability through various aggregating databases; (ii) the literature on ecological indicators; and, (iii), recent related developments such as the idea of species’ population “Essential Biodiversity Variables”. It should however also be applicable to primary research applications, including situations where trends are related to environmental drivers, either as continuous or as categorical variables (see the “Causal inference” section below for more on this point). A supporting glossary of statistical terms used here can be found below (Box 1); references to relevant risk of bias tools used in other disciplines are in separate supplementary material to the main paper.

## Structural outline

This guidance follows the general format of the recent update of the PRISMA tool for reporting systematic reviews and meta-analyses (Page et al., 2021); in that document, an explanation of the rationale behind each signalling question is given, followed by a concise summary of the expected response type from researchers. One small difference, however, is that we do not elaborate with model answers, but instead refer the reader to a worked example provided elsewhere in the supplementary material accompanying this publication. In the remainder of this introduction to the ROBITT tool, we restate some key concepts that should be kept in mind by researchers using this approach to assessing potential sampling biases in species occurrence and/or abundance datasets within the framework of temporal trend assessments.

## Conceptualising sampling biases and representativeness within the ROBITT framework

A key concept for ROBITT assessments is the recognition that sampling biases are a phenomenon emerging from the combination of a dataset, a research question, and some analytical attempt to answer that question. Strictly speaking, statements such as “this dataset is biased” do not make sense in isolation. Implicit in such statements is some larger statistical population (see Box 1 for a definition of the statistical population and other key terms used here) that is being used as a reference point by a researcher. The results from one analysis might be biased for the purposes of characterising a larger statistical target population, but might also be assessed to be a robust summary of some smaller population. Given this, researchers should strive for clarity of thought and exposition in describing biases *as they relate to their research question, chosen scale, and analytical approach*, and not merely implicitly, such that it is assumed that the statistical target population sampled should be obvious to the reader. Depending on the spatio-temporal scale of an analysis, some point of detail might be important for bias, whereas at another scale the same point might be irrelevant. For example, variability or unrepresentativeness in “visit”-level data (e.g., information on day- and small scale location-resolved species occurrence data recording events^^[[1]](#footnote-1)^^) might be very important to consider if one is making inferences, or attempting to extract information, at these scales; however, asking one’s research question at a larger spatio-temporal scale (e.g. because additional historic data were only available at such a resolution), and using different analytical techniques at this larger scale, might mean that potential biases in data at the finer scale alone are much less important, or even irrelevant (e.g. because they are aggregated over)^^[[2]](#footnote-2)^^. Such clarity of purpose in determining the relative importance of data structures for the potential for bias is one intended outcome of a ROBITT assessment—truly understanding the properties of the data available to answer a research question should result in more appropriate analyses, clearer, more comprehensive methods sections, and/or more clearly caveated conclusions. Whilst none of this should come as a surprise to researchers, arguably the easy access to “big data” in the modern ecological age, coupled with the pressure to publish, can lead to these considerations being overlooked or poorly reported (e.g. Fraser et al., 2018).

Box 1. Glossary of relevant terms

| **Bias (in general):** A systematic deviation from the truth in data acquisition, analysis, interpretation, publication etc. Due to the many ways in which such deviations might occur, some disciplines have created catalogues of biases in order to support clarity of communication and focus between researchers (e.g. see <https://catalogofbias.org/> for health research).  **Bias (in frequentist statistics):** The tendency of a sampling distribution-based estimator to under- or overestimate the true population value of a parameter.  **Bias (in sampling):** Where samples do not match the population in important characteristics (i.e., the sample is not representative of the population). Note the difference between estimator bias in frequentist statistics and sampling bias: frequentist bias is a property evaluated over multiple samples, whereas sampling bias, in the more general sense of a failure of representativeness, can be defined in the context of a single sample. A small sample may be unrepresentative even though it is a true probability sample (although some statistics may take this into account through increased confidence interval widths or similar).  **Convenience sampling:** A form of nonprobability sampling, whereby units are selected because they are easily accessible. For example, a naturalist might record sightings in their back garden, or near a road, rather than travelling to a remote location. This results in a convenience sample; also sometimes called opportunistic sampling.  **Probability sampling:** Sampling from a statistical population using probability theory; that is, random sampling of some form. Results in probability samples which will be representative of the statistical population on average.  **Nonprobability sampling:** Sampling from a population whereby each member of that population does not have an equal chance of selection (or an equal chance conditional on some set of weights). Results in nonprobability samples which may be unrepresentative of the population in ways that could be important for resulting inferences if there are correlations between the selection mechanism and the properties of the population relevant to the research question (Meng, 2018).  **Parameter:** A (statistical distribution-based) estimate of some quantity pertaining to a statistical population (e.g., its mean, variance, etc.).  **Statistic:** An estimator of a population parameter derived from a sample of that population. (Note that a parametric model is implied by this definition).  **Statistical inference:** The process of drawing inferences about a statistical population from a sample (or set of samples) from that population; the inferences made from any given sample will also depend on the study goals (e.g. prediction, causal understanding, description; Shmueli, 2010) and the statistical model or models used to investigate these (Barnett, 1982). (Note that here we distinguish description, as one possible inferential goal, from descriptive statistics, which are normally defined as simple descriptors of a sample).  **Statistical population:** The complete set of units, with some identified properties, of which a sample is desired. For example, the true presence/absence status of all vascular plants in 1 km land-containing grid cells in a country. Sometimes also called the target population, or simply “population”. We avoid the latter term to avoid confusion with the common use of the term in ecology to refer to species’ populations.  **Representativeness:** The degree to which a sample matches the population of interest in terms of features that are important for inference. This may apply to multiple domains; for example, a sample may be representative of the geographic domain of the population, but not the taxonomic domain. Representative samples are best obtained by probability sampling of a population. Unrepresentativeness is also referred to as **“coverage error”** in some disciplines (as distinct from the frequentist’s concept of confidence interval coverage).  **Validity:** Validity is the degree to which a study’s conclusions are likely to be true of the statistical population about which inferences are desired. This may be decomposed into internal and external validity. **Internal validity** is the extent to which the desired inferences can be accurately extracted from the sample, e.g., whether the effect of some intervention is well-identified from some experimental or quasi-experimental approach. **External validity** is the degree to which the results may or may not generalise to the wider population of interest. The concepts of internal and external validity are most frequently encountered in the context of causal inference, where the main aim is typically to estimate the effect of some intervention, e.g., the effects of some new medical treatment or social policy; however, they also apply to descriptive and predictive research. For example, describing national-level temporal trends in a species from a very unrepresentative sample would have low external validity, even if the data themselves were collected in a very robust fashion from the sampled locations (i.e. with high internal validity). In relation to any clearly stated inferential goal then, low and high validity map to high and low risks of bias respectively. |
| --- |

It is worth emphasising here that incomplete or uneven coverage of any research question-relevant domain (e.g. geographical, environmental, taxonomic etc.) does not necessarily equal bias relative to an inferential goal. First, researchers should recall that inference relates to their research question, and representative sampling of some of the domains assessed here may be irrelevant to their goals (this should be clarified through answering questions 2.1, 2.2, and 3.1 of ROBITT). For example, one would not expect a researcher’s data to be representative of some geographical area if they had stated that they were specifically interested in some area of environmental space. Users should not feel compelled to explain how they would mitigate uneven coverage in any domain unless it has the potential to bias their conclusions. Second, it is also possible that homogeneity of some ecological state or relationship across some domain means that sampling does not, at least in theory, need to be either extensive or random to capture accurate parameter estimates. A caveat, however, is that this argument cannot normally be proven without separate unbiased samples, or a full census, and therefore should not be claimed without very good supporting evidence. Third, there is the challenge of distinguishing between unrepresentative and representative sampling when the total extent of the statistical population is unknown (or subject to some uncertainty). For example, presence-only data on some taxon’s distribution may perhaps be representative of its area of occupancy, but the lack of samples from other parts of a geographic domain may mean that this cannot be known from the dataset alone with any certainty. Ultimately, the reliability of our samples as descriptions of reality can only be subjectively assessed (if we could access all data, i.e. reality, then assessment of representativeness would be a moot point), and expert knowledge and other available data or models will be required to come to a conclusion about sampling bias (Peters, 1991). If we truly cannot reach a conclusion about potential biases due to a fundamental lack of knowledge about the statistical population of interest, then simple descriptions of samples (i.e. descriptive statistics), rather than inferential statistics, will likely be more appropriate.

## Research quality and risk of bias

In other literature on study validity, efforts have been made to distinguish more general conceptions of research “quality” from risk of bias assessments. Indeed, as we discuss in the main manuscript, Sterne et al. (2016) noted that, in studies of healthcare interventions, a “shift in focus from methodological quality to risk of bias has been accompanied by a move from checklists and numeric scores towards domain-based assessments in which different types of bias are considered in turn.” Specific tools in healthcare research have attempted to provide a clear distinction between the concepts of study quality and risk of bias (see also supplementary material 5). For example, the GRADE framework (Grading of Recommendations, Assessment, Development and Evaluations), for developing and presenting summaries of evidence for making clinical practice recommendations in healthcare research, is supported by several full papers providing descriptions of the different quality areas assessed (e.g. see <https://bestpractice.bmj.com/info/toolkit/learn-ebm/what-is-grade/>). Risk of bias (Guyatt et al., 2011a) and imprecision (Guyatt et al., 2011b) are explicitly distinguished within this approach, with precision in GRADE focusing mainly on the examination of 95% frequentist confidence intervals and study power. Overall, the GRADE system was erected to examine a body of evidence at the level of outcomes under interventions across studies, rather than at the study level, and assessments of the five GRADE areas of risk of bias, imprecision, inconsistency (across studies), indirectness (a type of low external validity), and publication bias are presented within GRADE in this context. Some of the ROBITT questions (e.g. 2.3 and 2.4) arguably address aspects of quality other than risk of bias; however, the focus on such issues within the ROBITT tool (e.g. data provenance, cleaning etc.) should always be considered in the service of the risk of bias question, the inferential aims of the research, and the model (or other approaches) used to address these. Another challenging aspect here is the distinction between precision and bias at the single study level: whilst the imprecision area of GRADE focuses on power and confidence interval width, we suggest that the type of research addressed by ROBITT would typically be more concerned with descriptive inference than with hypothesis testing.^^[[3]](#footnote-3)^^ Averages taken across small probability samples may be theoretically unbiased in the long run in the context of a frequentist estimator, but, for the purposes of accurate description, such samples may still be individually very unrepresentative (although particular statistics, at least for probability samples of well-defined target populations, may take this into account through increased confidence interval width or similar). For the types of aggregated data primarily targeted by ROBITT, we therefore consider imprecision here in the context of sampling biases, rather than attempting the distinction between imprecision and risk of bias used by the GRADE system.

## Causal inference

Much of the risk of bias literature in other disciplines focuses on the effects on interventions (e.g. see supplementary material 5). In this type of research, the questions asked are typically explicitly causal, because the desired inference concerns whether some action results in some outcome. This has also been the typical focus of evidence-based conservation, at least historically (e.g. Lortie et al., 2015). ROBITT is primarily focused on descriptive inference of the type that is often used for ecological indicators (e.g. van Strien et al., 2009), and, more recently, by the Essential Biodiversity Variable literature (Jetz et al., 2019). However, the distinction is not absolute, and there are many examples of ecological studies that use aggregated, or other, species occurrence/abundance data in attempts to reach causal conclusions. For example, Woodcock et al. (2016) split wild bee occurrence data for Britain into two spatial subsets based on the agricultural use of neonicotinoid insecticides, assessing occupancy temporal trends for the taxa concerned in each subset. Whilst this type of assessment is correlative, there is often clearly a causal motivation behind the research (e.g. the title of Woodcock et al. 2016 certainly implies causality). Whilst the ROBITT tool has not been designed to explicitly deal with these types of situations, we suggest that the tool will still be useful when researchers are attempting to make causal inferences from such observational data. In the example just given (Woodcock et al., 2016), the domain representativeness of the data in the two spatial subsets could have been assessed separately in order to investigate the potential for confounding; additionally, the full dataset could also have been assessed in order to investigate its external validity. In the future, we anticipate that other risk of bias tools will be developed within ecology to fully meet the needs of causal inference research (again, see supplementary material 5 for potential models).

Completing the ROBITT form and the research process

Whilst the questions that constitute the ROBITT form require individual answers, it may be the case that researchers prefer to provide such responses in the main text of a research paper or report. This is not a problem; as a point of comparison, PRISMA 2020 (Page et al., 2021) provides a checklist format that allows researchers to direct the reader to the answer to any given question. This could also be the case with ROBITT; for example, clear subheading or section references could be provided in response to a question, provided it was completely clear that the manuscript text referenced was a clear and complete answer to that question; referencing version-controlled code canprovide the required information. Cross-referencing within the form may also provide sufficient information; for example, it is possible that answers provided under question 2.2 might also be adequate for answering questions 3.11 and/or 3.12, or vice versa. Ultimately, researchers should assess whether the intended logic of their research programme and answers is clear, overall, from the responses provided.

We suggest that researchers will get the greatest benefit from the ROBITT tool if they use it to structure their research. The questions contained in the tool are questions that researchers should be asking themselves regardless; indeed, ROBITT provides an opportunity to demonstrate the large amount of preparatory work that typically goes into studies of temporal trends in biodiversity, but which may not always be reported in research papers. If, on the other hand, a ROBITT form is completed just before, say, the submission of an article for publication, then it may reveal problems that could otherwise have been dealt with earlier. Completing the ROBITT form during the research process therefore has the potential to save researchers' time. We are not suggesting that all studies of temporal trends in biodiversity should be centered on a ROBITT form, but we do consider that it will provide a useful framework for structuring the required thought processes in many cases, particularly where aggregated and/or observational data are involved. The requirement for researchers to carefully document the limitations of their work should result in positive outcomes for all scientists, especially in terms of thinking more clearly about how to approach, and model, datasets that may be seriously biased in relation to some inferential goal, and also in highlighting domains where data are severely lacking.

In some cases completing a ROBITT form may be an iterative process. For example, researchers might complete a first iteration of the tool and find that data coverage is not sufficient in portions of their geographic domain of interest. In this case, a researcher might decide to redefine their geographical domain to exclude the poorly sampled regions; this would mean completing a second iteration of the ROBITT form using the appropriate subset of the data. If users become aware that their data do not permit inferences across their desired domains midway through completing an iteration of the ROBITT form, there may be no need to continue with that iteration. Instead, they might decide to redefine their domain as required and move onto the next iteration without answering every question. Nevertheless, where multiple iterations are needed, the user should clearly version control (i.e. tracking and recording changes over time) their documents and provide this history as supporting information to their studies. Such an approach could also fulfil the requirements of preregistration where this was relevant for a particular analysis (e.g. where *p*-values were being used for Neyman-Pearson-type inference with a preset alpha). Existing tools such as the Open Science Foundation infrastructure ([www.osf.io](http://www.osf.io)) or GitHub ([www.github.com](http://www.github.com)) could be used in order to capture all versions of the ROBITT form used, and to avoid accusations of data-dredging or forking paths (i.e. changing models or other aspects of the analytical process based on looking at the data, but using inferential frameworks that assume that analytical plans have been specified *a priori*).

Whilst users may go about answering the questions in ROBITT in the best way they see fit, we note that the use of heuristics to indicate the potential for bias will often be a useful way to supplement text-based responses. We use the term “heuristic” to acknowledge that it is not possible to quantify the true extent of any biases without probability samples for comparison. There have been many heuristics used to screen biodiversity data for biases in the literature (e.g. see Boyd et al., 2021). We briefly review these heuristics in Table 1.

Table 1. A non-exhaustive list of heuristics used to assess biodiversity data for potential biases in the geographic, environmental and taxonomic domains.

| **Domain** | **Heuristic** | **Details** |
| --- | --- | --- |
| Geographic | Map of the density of records or sampling locations across the geographic domain | This is the simplest and most common way to assess geographic coverage in a dataset. |
|  | Multiple maps showing temporal variation in geographic coverage of records or sampling locations | Multiple maps, each pertaining to some time period, allow one to assess whether the distribution of sampling or records has changed over time (e.g., Nøttestad et al. 2015) |
|  | Nearest neighbor index | Ratio of the average nearest neighbor distances of the empirical sample to average nearest neighbor distances of a random distribution, or a simulated random distribution (Clark & Evans, 1954). The quotient indicates the degree to which the data depart from a random distribution. This heuristic is best applied to presence-absence or abundance data, where the distribution of sampling is known. However, it may also be informative about biases in presence-only data when those data are aggregated to the “target group” level (Phillips et al., 2009). |
| Environmental | Map of the distribution of records in some dimension of environmental space | Many authors have mapped their data in environmental space, whether in one dimension (e.g., along a temperature gradient; Speed et al. 2018) or many dimensions. Where more dimensions are considered (e.g. several climate variables), ordination techniques may be used to summarise environmental space (Pescott et al., 2019b). |
| Taxonomic | Inventory completeness | Inventory completeness is the proportion of species known to occur in some spatio-temporal unit that have been recorded (e.g., Shirey et al., 2021). This is a measure of taxonomic coverage. It can be calculated for different time periods and spatial units to look at spatio-temporal variation in taxonomic coverage. |
|  | Congruence of species’ range size and the number of times they have been recorded | One way to estimate whether rare taxa are oversampled relative to commoner ones is to look at their deviation from a fitted relationship between range size and the number of records across taxa (Barends et al., 2020; Speed et al., 2018). |

## Future updates

The version of ROBITT presented in this paper can likely be improved in the future, and we welcome feedback from all who use it. We expect to publish periodic updates to the tool and guidance, and add additional examples, in order to increase its usefulness and clarity over the coming years.

1. Iteration

**1.1 ROBITT iteration number**

| **Iteration** | **Comments** |
| --- | --- |
|  |  |

**Explanation:** It is possible that the completion of a ROBITT form will indicate that the available data do not permit one’s desired inferences. In this case, the user might redefine their statistical population and/or research question and decide to complete a second iteration of the form etc. to assess the data in relation to these. Here, the user should state whether this is the first iteration of their ROBITT form, or whether it reflects a refined research goal and statistical population.

*Essential elements:*

- A statement of the iteration number.
- A brief explanation of how your inferential goal or statistical population has been refined since the previous iteration.
- A link to the previous version where possible.

1. Research statement and pre-bias assessments

Statistical population of interest

**2.1 Define the statistical target population about which you intend to make inferences.**

| **Domain** | **Extent** | **Resolution** |
| --- | --- | --- |
| Geographic |  |  |
| Temporal |  |  |
| Taxonomic (or other relevant organismal domain such as functional group etc.) |  |  |
| Environmental |  |  |

**Explanation:** In order to assess the representativeness of your sample, the statistical population that you intend to make inferences about should be clearly defined. This question is therefore designed to lead to clear statements about the target population that will be assessed throughout the remainder of the ROBITT tool, and which will be evaluated by editors, reviewers, and other future readers. Users will notice that this question duplicates elements of question 2.2 below, but the table format here is intended to act as a clearly stated “quick reference” summary of the basic elements of the domains of research interest.

Note that not all the domains listed in the table for the researcher to complete (i.e. geographical, temporal, taxonomic, environmental) will necessarily be relevant for all research questions. Researchers may sometimes be investigating trends (or non-temporal associations) across environmentally defined zones rather than geographic areas *per se*; it is up to the researcher to complete the rows of the table considered relevant to their question(s).

In some cases resolution and extent may be difficult to define for some domains. For example, taxonomic extent perhaps most intuitively refers to the coverage across a phylogenetic tree (i.e. horizontally), whereas resolution would refer to the granularity of this coverage (i.e. whether the researcher desires inferences at the species or some coarser level). For the environmental domain, extent and resolution will be dependent on how this is conceptualised in the first place. If some multidimensional space based on some ordination or other processing of multiple variables is used, then resolution might relate to the scale at which this has been created, and extent will be whichever part of this space is of interest. Approaches that conceptualise environmental space as some particular habitat or land cover are likely to be able to use definitions that are more straightforward (e.g. extent could merely be all woodland as mapped according to some land cover product). Ultimately researchers are free to complete the table as they wish, the key outcome is simply a clear statement of the statistical target population, rather than some universal codification of these categories across uses of the tool.

*Essential elements:*

- Clearly stated information on the desired extents (i.e. the areas or “spaces” for which inferences are intended to be valid) and resolutions (i.e. grain size or scale) for each relevant domain.
- If some domains are not relevant, statements should be provided explaining why.

Inferential goal

**2.2 What are your inferential goals?**

**Explanation:** This question is intended to elicit a clear statement about what you ultimately wish to infer regarding the statistical population defined in question 2.1 (note that “research question” might be considered a synonym for “inferential goal”, but we use the latter here to emphasise the link to statistical inference). For example, temporal trends in occupancy (for the extent and at the scale stated in 2.1) might be of primary interest. If the model or other framework that you have erected in order to reach your inferential goal includes other parameters that must be estimated (e.g. relating to detectability), then these could also be stated here; however, such model-specific detail could also be provided under questions 3.11 and/or 3.12 below.

*Essential elements:*

- A clearly stated inferential goal concerning the ecological state variable(s) of interest.

Data provenance

**2.3 From where were your data acquired (please provide citations, including a DOI, wherever possible)? What are their key features in respect of the inferential aims of your study?**

**Explanation:** Providing access to your data (ideally through an open and stable repository) will allow other researchers to investigate your conclusions and to build on your findings. If this is not possible for some legitimate reason, then clear descriptions of how the data were sourced should be provided, and the reason that data are not being shared clearly stated. In addition, describing the various attributes of your data that are considered important for addressing your inferential aims: (i) helps you to investigate and understand your data before starting to model or otherwise evaluate them; and, (ii), assists others in evaluating the appropriateness of any conclusions that you have reached. The data properties assessed and described here should be at a scale that is relevant for the inferential goals as described in 2.2. For example, descriptions of data properties that exist at a finer scale than one of the inferential domains, and which are not relevant to any model or analytical procedure used, may not be very useful to document (although the reasoning behind such an omission may itself be worth noting).

We expect that this section will allow researchers to demonstrate the extensive preparatory work that is often done in advance of analyses, but which sometimes goes unreported in manuscripts due to space limitations. Clearly there is potentially a large amount of information that could be reported here, which is why we emphasise that researchers are *not* being asked to document every single feature of their dataset, but, instead, are being asked to consider and understand specific features that may introduce important errors relative to their research question.

*Essential elements:*

- Report the data type. For example, data might be presence-only, presence/absence, counts etc. If some or all data are degraded from one type to another for the proposed analysis (e.g. count data converted to presence/absence for combination with another presence/absence dataset), then this should also be noted.
- Report the geographic, temporal, taxonomic, and/or environmental extents and resolutions of the data (these could be supported by plots and/or maps). This reporting should take into account any cleaning or manipulation steps reported in 2.4 below; before/after cleaning and manipulation comparisons may also be important to document here. Note that clearly distinguishing between apparent extent and actual data resolution may be important for your inferential goal(s): for example, a dataset may cover a range of years, but comparisons between time periods may be undermined if it is not recognised that some data points were originally poorly resolved and have been erroneously made more precise (e.g. species occurrence records with different start and end dates being assigned to a single day). Likewise, location data originally collected as a polygon or grid may have been reduced to a point (with or without the associated uncertainty) for databasing.
- Report anything that is known about the data collection process that is relevant to the inferential goal (2.2) and the model that will be used to evaluate this (e.g. see questions 3.11, 3.12 below). For example, it may be important to know whether data were opportunistically sampled by volunteers, or whether they were collected as part of a structured professional survey.
- If the data are from an aggregated database, then what is known, if anything, about the major constituent datasets contributing? Can you usefully break down any of the elements assessed here in terms of the proportions of the data with any given property? For example, some proportion of your data might have originally been collected, or subsequently summarised for databasing, according to some grid (i.e. lattice), whereas another proportion might be true point data with associated error radiuses. Researchers should highlight any such structure that is relevant to their statistical population and inferential question(s) (see 2.1, 2.2 above).

Data processing

**2.4 Provide details of, and the justification for, all of the steps that you have taken to clean the data described above.**

**Explanation:** Datasets typically require some processing before use in order to answer any given research question. In particular, biodiversity data that were not actually collected by the researchers using them for analyses may contain subsets that are inappropriate for the intended inferential use. For example, data available through data aggregation platforms such as GBIF may be resolved to a variety of geographic, temporal, and taxonomic scales, some of which may be too imprecise for stated inferential goals (see 2.1, 2.2 above). As we have highlighted elsewhere, bias as assessed through this tool is an emergent property of an attempt to answer a specific research question using a particular dataset and some analytical approach. Whilst potential errors and sources of imprecision in a dataset will not necessarily lead to bias in the sense of a systematic deviation from the truth, in our experience data processing steps have a very high potential for decreasing the representativeness of datasets, therefore the clear documentation of these steps (e.g. with links to reproducible code where possible), and the assessment of the potential risk of bias *after* such processing has been performed (section 3 below), are both essential.

*Essential elements:*

- Report any rules that were used to exclude, process, or correct data within your study. For example, geographically, temporally, and/or taxonomically imprecise or erroneous data points may have been removed or adjusted. Links to code can be provided here if relevant.

1. Bias assessment and mitigation

This section begins with a specification of the geographic (i.e. spatial), temporal, and taxonomic resolutions (i.e. grain sizes or scales) at which the bias assessment will be conducted. Generally, these should match the resolutions at which inferences are desired (as specified in the research statement section above).

Assessment resolutions

**3.1 At what geographic, temporal and taxonomic resolutions (i.e. scales or grain sizes) will you conduct your bias assessment?**

**Explanation:** Generally, the geographic and temporal resolutions of your bias assessment should match the resolutions at which inferences are desired (as specified in section 2 above); for example, it would not make sense to conduct your bias assessment on a dataset that was subsequently reduced further through some data cleaning process (see 2.4 above). Likewise, for some research question/analytical model combinations, the scale of the analysis in some domain may mean that some aspects of the data are irrelevant for bias (e.g., as we note in the introductory section “Conceptualising sampling biases and representativeness within the ROBITT framework” above, biases in small-scale “visit” data may not be relevant if analyses are to be conducted at much larger spatio-temporal scales; e.g. Hill, 2012). Ideally, the taxonomic resolution should also match the resolution at which inferences are desired, however, this may not always be possible. For example, if your species data are presence-only data, a frequently used approach is to infer information on sampling effort in locations where the focal taxa was not recorded from records of similar taxa.^^[[4]](#footnote-4)^^ Or, to assess taxonomic biases, it might be necessary to compare some aspect of the data collection process across species or other taxonomic groups.

*Essential elements:*

- A set of clearly stated resolutions (i.e. scales or grain sizes) for your bias assessments.

Geographic domain

**3.2 Are the data sampled from a representative portion of geographical space in the domain of interest?**

**Explanation:** Whilst the temporal element is primary for the ROBITT assessment (unless perhaps the form is being used for assessing a purely spatial research question, such as the creation of a Species Distribution Model)^^[[5]](#footnote-5)^^, it is also useful to present an overall assessment of the available data with respect to each of the inferential domains. In studies of temporal trends in biodiversity, it is not uncommon for researchers to exaggerate the geographical extent over which their conclusions are relevant (see e.g. Cardinale et al., 2018). To reduce the potential for such misleading claims, this question requests an overall presentation of the available data in relation to the overall geographical domain of interest. Note that it is always possible that your data are not representative of the geographic domain of interest (e.g. a country) but are nevertheless representative of the distributions of some or all of the focal taxa (e.g. no records of some species where it does not occur). If you suspect that this is so, can you provide evidence that this is the case (e.g. from expert opinion)? Otherwise, you must explain how you will reliably extrapolate from sampled to non-sampled locations in the relevant bias mitigation section (3.4), or clearly restrict the scope of your inferences.

*Essential elements:*

- A clear summary and demonstration (e.g. using a map or summary statistics) of how far your data are representative of your geographical domain of interest.
- If the representativeness of certain subsets of your data is assumed based on available ecological knowledge, even though you lack coverage of the entire domain, please provide evidence supporting these claims.

**3.3 Are your data sampled from the same portions of geographic space across time periods?**

**Explanation:** For robust inference concerning temporal trends within your geographic domain of interest, it is typically necessary that the available data are sampled from the same portion of geographic space over time. For example, a species might be doing well in one part of a country, and less so in another. If the data are sampled from the former portion of the country in one time period, and the latter portion in a second period, then one might come to the artefactual conclusion that the species is in decline when in fact this simply reflects a sampling bias relative to the desired inferences. There will be some exceptions to this, but these should be clearly explained and documented. For example, if there was strong evidence that the most important drivers of change in your ecological state variable of interest were uniform across your geographic domain, then unrepresentative coverage across time periods might be tolerated.

*Essential elements:*

- A clear summary and demonstration of how far your data are representative of your geographical domain of interest across your time periods of interest.
- If it is not the case that data are consistently representative of your geographic domain over time, then please attempt to explain the reasons for this (e.g. large increases in recording/reporting effort over time that has resulted in large changes in sample representativeness of this domain).
- If there is variation in geographical representativeness across time, but this is desirable (or assessed to be not relevant) for your research question of interest, then please provide information on this assessment here.

**3.4 If the answers to the above questions revealed any geographic biases, or temporal variation in geographic coverage, please explain, in detail, how you plan to mitigate them.**

**Explanation:** Identified geographic biases that are relevant for your research question should be mitigated, or clearly acknowledged in terms of their likely impact on your inferences. Mitigating action could include restricting the extent or resolution of your inferences (which, in the most extreme case, could mean deciding not to proceed with the planned analysis without collecting more data); modifying the data (e.g. spatial thinning or weighting); or attempting to adjust for the biases as covariates in some model-based approach. This is not an exhaustive list of potential bias adjustments, and other strategies may be available. Researchers should try to specifically explain how any proposed mitigating action will account for a particular bias; do not merely make broad or generic statements (such as, for example, “site-occupancy modelling was used to deal with biases”) that do not clearly link a particular potential bias to a specific attempt to correct for it at a relevant scale. Finally, see the general points concerning the assessment of potential sampling biases made above under the introductory section “Conceptualising sampling biases and representativeness within the ROBITT framework”.

*Essential elements:*

- A clear list of identified potential biases in the geographic domain (including over time), along with explicit documentation of how each potential bias is to be dealt with through your analytical approach(es).
- If no mitigating strategy can be identified, then this section should detail how and where your paper or other research report clearly limits the scope of its inferences, with particular focus paid to items such as titles, abstracts, highlights and conclusions, all of which are regular locations for inferential “over-reach” in published research.
- If bias mitigation strategies of some sort are to be used, then sensitivity analyses are also likely to be extremely useful for exploring the impacts of potential biases on conclusions.

Environmental domain

**3.5 Are your data sampled from a representative portion of environmental space in the domain of interest?**

**Explanation:** It is possible that your data are representative of geographic space in your domain of interest, but are not representative of the range of environments in that domain at some scale. For example, your data may appear to be randomly distributed across some country, but on closer inspection it could become apparent that they are all sampled from lowland areas, or areas with above average precipitation. If focal taxa fare well in lowland conditions, or poorly where rainfall is high, then the resultant conclusions about trends in those species’ abundances or distributions might be biased.

In answering this question you should attempt to assess whether your data are representative of environmental space in your geographic and temporal domains of interest, or, if applicable, of your environmental domain as specified in question 2.1. This should begin with a definition of your environmental domain (e.g., climatic, land cover, or other environmental variables relevant to your statistical population and inferential goal); note that this is likely to be a multidimensional space, and, as such, may be considered to be a species’ niche for some research questions. If you have defined your statistical population in an environmental domain in question 2.1, then it is clearly important that your data are representative of that domain; otherwise, they will not be representative of your target population. Even if you did not define your statistical population in some environmental domain (say, because your inferences pertain to geographic areas) it might still be important to understand whether your data are representative of environmental space in the geographic and temporal domains of interest. For example, if your data were collected in some portion of environmental space that the focal taxon favors (or avoids), then any estimates of population trends might be quite unrepresentative of the average trend in the geographic domain. Note that this domain could include things like site selection bias (e.g. monitoring species only at the sites with the largest populations, or only sampling near roads or in protected areas), because these can often be linked to aspects of the environment that are being over- or under-sampled. However, such phenomena may sometimes be best discussed under question 3.11 below if it is not clear how particular sampling biases actually relate to environmental space.

The ease with which you are able to obtain the environmental data to answer this question may depend on the temporal extent of your study. If you are looking at trends over recent decades, then this might not be an issue. Global land cover and climate data are available at increasingly fine spatial and temporal resolutions, and with increasing accuracy. If you intend to draw inferences about the distant past, however, then it will likely be more difficult to obtain environmental data, if it is possible at all (but see e.g. Goldewijk et al., 2011). In this case, you might need to make additional assumptions to estimate the extent to which your data are environmentally representative. One option is to use more recent environmental data aggregated to some coarse spatio-temporal resolution. For example, you could use the 19 worldclim bioclimatic layers (Fick and Hijmans, 2017) which represent averages over the period 1970–2000 at, say, a spatial resolution of 1°. Of course, some environmental variables, such as elevation and photoperiod, are likely to have remained roughly constant over time. Where researchers want to measure change in the distant past, it might be most appropriate to assess the representativeness of the data with respect to these variables. Indeed, these variables might well be indicative of more relevant climate conditions; for example, it tends to be cooler at higher altitudes, etc. However, ultimately, the definition of an environmental domain is only ever going to be as useful as the existing datasets that are relevant to the inferential question; as with any research relying on observational data, the confounding of available data with unavailable, unmeasured, or unknown environmental variables may seriously affect our true understanding of a system.

*Essential elements:*

- A clear summary and demonstration of how far your data are representative of your environmental domain of interest, or of environmental space in your geographic domain of interest.
- If the representativeness of historic portions of your data is assumed based on more recent environmental data, please be explicit about this.
- (See ‘Geographic domain’ above for additional points that may also be relevant here).

**3.6 Are your data sampled from the same portion of environmental space across time periods?**

**Explanation:** Uneven sampling of environmental space over time can bias estimates of temporal trends in species’ populations. For example, if environments that a species favors are sampled in the early periods, then environments that it does not favor are sampled in the later periods, then one might come to the artefactual conclusion that the species is in decline. Of course, it is important to remember that relevant aspects of the environment, in any given geographic domain, may themselves have been changing over time. Hence, if the same parts of geographical space have been sampled over long time periods, then the portion of environmental space sampled may vary. If one simply wants to understand how some aspect of biodiversity has changed over time in some geographic region, then it might not matter whether the same portion of environmental space has been sampled over time (indeed, environmental change could well be the driver of those changes). In this case, it will likely be more appropriate to assess spatial coverage over time in the geographic domain, ideally whilst also attempting to understand how the sampled environmental space has changed relative to that of the entire geographic domain. As with many parts of the ROBITT process, and indeed with robust inference generally, the key here is clearly specifying one’s research question, and robustly interrogating data to understand the statistical population sampled in all of its relevant domains. In a similar fashion to our final point under the “explanation” section of 3.5 above, developing such understanding of how a (changing) environmental domain has been sampled over time may sometimes be severely limited by available environmental data.

*Essential elements:*

- A clear summary of which portions of your environmental domain have been sampled over time.
- If there is variation in environmental representativeness across time, but this is desirable (or assessed to be not relevant) for your research question of interest, then please provide information on this assessment here.
- An acknowledgement of any assumptions made in the absence of historic environmental data.

**3.7 If the answers to the above questions revealed any potential environmental biases, or temporal variation in environmental coverage, please explain, in detail, how you plan to mitigate them.**

**Explanation:** Identified environmental biases that are relevant for your research question should be mitigated, or clearly acknowledged in terms of their likely impact on your inferences. Mitigating action could include restricting the extent or resolution of your inferences (which, in the most extreme case, could mean deciding not to proceed with the planned analysis without collecting more data); modifying the data (e.g. spatial thinning or weighting); or attempting to adjust for the biases as covariates in some model-based approach. This is not an exhaustive list of possible bias adjustments, and other strategies may be available. Researchers should try to specifically explain how any proposed mitigating action will account for a particular bias; do not merely make broad-brush statements (such as, for example, “site-occupancy modelling was used to deal with biases”) that do not clearly link a particular potential bias to a specific attempt to correct for it at a relevant scale. Finally, see the general points concerning the assessment of potential sampling biases made above in the introductory section “Conceptualising sampling biases and representativeness within the ROBITT framework”.

*Essential elements:*

- A clear list of identified potential biases in the environmental domain, or in the environmental space associated with the geographic and temporal domains, along with explicit documentation of how each potential bias is to be dealt with through your analytical approach(es).
- If no mitigating strategy can be identified, then this section should detail how and where your paper or other research report clearly limits the scope of its inferences, with particular focus paid to items such as titles, abstracts, highlights and conclusions, all of which are regular locations for inferential “over-reach” in published research.
- If bias mitigation strategies of some sort are to be used, then sensitivity analyses are also likely to be extremely useful for exploring the impacts of potential biases on conclusions.

Taxonomic domain (or other organismal domain, e.g., phylogenetic, trait space etc.)

**3.8 Is the sampled portion of the taxonomic (or phylogenetic, trait or other space if more relevant) space representative of the taxonomic (or other) domain of interest?**

**Explanation:** In studies of temporal trends in ecology, researchers sometimes claim that their inferences apply to some wider taxonomic grouping when in fact the data pertain only to a subset of this group (e.g. see the main ROBITT paper). This can lead to spurious conclusions because different species may respond differently to the same pressures. In answering this question, you should first be explicit about the number of species for which you have data, and, where possible, what proportion of the taxonomic domain as specified in question 2.1 these species account for.

It is possible that, despite a large proportion of species in your taxonomic domain appearing in your dataset, they are not sampled in proportion to their abundance (or frequency at some larger scale) in nature. It can be difficult to know whether species are sampled in proportion to their commonness within any time period, but please provide any information here that might indicate whether this is the case. Possible options include comparing species’ recorded range sizes (perhaps in units of grid cells) with the number of times they have been recorded, or seeking expert advice. Unrepresentativeness in this area may also be due to interactions between some aspect of the biodiversity under consideration and the geographic areas or environmental spaces sampled over time, e.g. perhaps urban areas, and therefore anthropophilic species, are better represented in some time periods than others.

If your statistical population is defined in some organismal space other than taxonomy (e.g., trait or functional space), the above points still apply. If you are interested in trends in pollinator diversity, for example, it is crucial to understand what proportion of pollinators in the given geographic and temporal domains are represented in your data.

*Essential elements:*

- A clear statement of the degree to which the taxa (or other organismal axis) represented in your data are representative of the taxonomic (or other organismal) domain as defined in question 2.1.
- (See ‘Geographic domain’ above for additional points that may also be relevant here).

**3.9 Do your data pertain to the same taxa/taxonomic domain across time periods?**

**Explanation:** Temporal variation in taxonomic coverage can bias estimates of trends in biodiversity. For example, one might want to estimate trends in insect populations in Europe from 1970 to present day. If monitoring schemes were established for particular insect groups (e.g., bees, hoverflies) at some point over this time period, then these groups may become more prominent in the data in later years. This could lead to the artefactual conclusion that bees and hoverflies are faring well when in fact they have simply been recorded relatively more often than other insect groups in recent time periods. As with the other domains above, if biological processes of relevance to the inferential question have altered the domain over time (e.g. some species are reliably known to have gone extinct, or to have colonised the area, over the time period of interest), then note this, providing evidence where required.

*Essential elements:*

- A clear demonstration of whether, and to what extent, the composition of taxa (or other relevant organismal quantity) in your data varies over time.
- If there is variation in taxonomic representativeness across time, but this is desirable (or assessed to be not relevant) for your research question of interest, then please provide information on this assessment here.

**3.10 If the answers to the above questions revealed any potential taxonomic biases, or temporal variation in taxonomic coverage, please explain, in detail, how you plan to mitigate them.**

**Explanation:** Identified taxonomic biases that are relevant for your research question should be mitigated, or clearly acknowledged in terms of their likely impact on your inferences. Mitigating action could include restricting the extent or resolution of your inferences (which, in the most extreme case, could mean deciding not to proceed with the planned analysis without collecting more data); modifying the data (e.g. spatial thinning or weighting); or attempting to adjust for the biases as covariates in some model-based approach. This is not an exhaustive list of potential bias adjustments, and other strategies may be available. Researchers should try to specifically explain how any proposed mitigating action will account for a particular bias; do not merely make broad-brush statements (such as, for example, “site-occupancy modelling was used to deal with biases”) that do not clearly link a particular potential bias to a specific attempt to correct for it at a relevant scale. Finally, see the general points concerning the assessment of potential sampling biases made above under the introductory section “Conceptualising sampling biases and representativeness within the ROBITT framework”.

*Essential elements:*

- A clear list of identified potential biases in the taxonomic domain, along with explicit documentation of how each potential bias is to be dealt with through your analytical approach(es).
- If no mitigating strategy can be identified, then this section should detail how and where your paper or other research report clearly limits the scope of its inferences, with particular focus paid to items such as titles, abstracts, highlights and conclusions, all of which are regular locations for inferential “over-reach” in published research.
- If bias mitigation strategies of some sort are to be used, then sensitivity analyses are also likely to be extremely useful for exploring the impacts of potential biases on conclusions.

Other potential biases

**3.11 Are there any other potential temporal biases in your data that relate to variables other than ecological states?**

**Explanation:** ROBITT as a whole has been designed to highlight potential biases in temporal trends, and all domains should be assessed with a view to evaluating the representativeness of the available data over time (see 3.3, 3.6, and 3.9 above). However, depending on your research question, and the resolution(s) at which this is being posed (see 2.1, 2.2, and 3.1 above), assessments of temporal biases in other properties may also be required. One way of conceptualising the difference between this section and the assessments of the preceding domains (geographic, environmental, and taxonomic) is to consider the difference between the representativeness of ecological state variables (such as abundance and occupancy), and biases in the estimation of observation process-related parameters (i.e. modelled elements related to measurement error). For example, site-occupancy models (Kéry & Royle, 2016) require estimates of detectability, typically based on repeated, small-scale, site visit data within particular time windows (“closure periods”); however, such estimates can themselves be based on unrepresentative data, and this can ultimately lead to biased inference (e.g. Royle, 2006). This section, then, is an opportunity for the analyst to consider temporal variation in their data that has the potential to lead to biases in model parameters that could subsequently lead to biased inference concerning ecological state variables of primary interest.

*Essential elements:*

- A clear description of temporal variation in particular data structures that are important for accurate inference, conditional on the model, or other analytical approach, that you are using.
- If some part of your model (e.g. some measurement error or observation process) is intended to address some issue with potential biases in the data (e.g. detectability), you should explore the potential consequences of the assumptions underlying these models of reality being violated.^^[[6]](#footnote-6)^^

**3.12 Are there any other potential biases not covered by the above questions that might cause problems for your inferences?**

**Explanation:** This question has been provided to cover any other potential biases that may not obviously fit within any of the preceding questions. Examples include variation in species’ baselines, phenology, time of day, recorder identity etc. (e.g. see Didham et al., 2020). Some of these may be more clearly dealt with in section 3.11 in relation to specific model types, or in other sections; however, listing such potential issues here will be useful for editors, reviewers, and other readers wanting to make their own assessments of whether any of this described variation is likely to affect any inferential claims arising from your research.

*Essential elements:*

- Clear descriptions of sources of other potential variation in your dataset that could undermine your inferential claims if not mitigated or clearly explained to the reader.

**3.13 If questions 3.11 or 3.12 revealed any important potential biases, please explain, in detail, how you will mitigate them.**

**Explanation:** As with the bias assessments of the fundamental domains listed above, this section is to provide you with the opportunity to detail how any other biases identified will either be mitigated or acknowledged.

*Essential elements:*

- A list of the identified additional biases and how they will be mitigated or highlighted to the reader. This may include model-based, or other analytical, adjustments; decisions to work at different scales; and/or in-text acknowledgements of important sources of bias that are not easily adjusted for. Note that, as with all remaining biases, important research summaries, such as paper titles, abstracts, and conclusions, should not claim or imply more certainty or reach than is the case based on this bias assessment.

1. Guidance references

Ball-Damerow, J. E., Brenskelle, L., Barve, N., Soltis, P. S., Sierwald, P., Bieler, R., ... and Guralnick, R. P. 2019. Research applications of primary biodiversity databases in the digital age. *PloS ONE*, 14(9), e0215794.

Barends, J. M., Pietersen, D. W., Zambatis, G., Tye, D. R. C., & Maritz, B. 2020. Sampling bias in reptile occurrence data for the Kruger National Park. *Koedoe*, 62, 1–9. https://doi.org/10.4102/koedoe. v62i1.1579

Boyd, R.J., Powney, G.D., Carvell, C. and Pescott, O.L., 2021. occAssess: An R package for assessing potential biases in species occurrence data. *Ecology and Evolution*, 11(22), 16177-16187.

Cardinale, B.J., Gonzalez, A., Allington, G.R.H. and Loreau, M. 2018. Is local biodiversity declining or not? A summary of the debate over analysis of species richness time trends. *Biol. Conserv.* 219, 175–183. doi:10.1016/j.biocon.2017.12.021

Clark, P., & Evans, F. 1954. Distance to nearest neighbour as a measure of spatial relationships in populations. *Ecology*, 35, 445–453. https://doi.org/10.1007/BF02315373

Didham, R. K., Basset, Y., Collins, C. M., Leather, S. R., Littlewood, N. A., Menz, M. H., ... and Hassall, C. 2020. Interpreting insect declines: seven challenges and a way forward. *Insect Conservation and Diversity*, 13(2), 103-114.

Fick, S.E., Hijmans, R.J., 2017. WorldClim 2: new 1-km spatial resolution climate surfaces for global land areas. Int. J. Climatol. doi:10.1002/joc.5086

Fraser, H., Parker, T., Nakagawa, S., Barnett, A. and Fidler, F. 2018. Questionable research practices in ecology and evolution. *PLoS ONE*, 13(7), e0200303.

Gelman, A. and Loken, E. 2014. The statistical crisis in science: data-dependent analysis--a" garden of forking paths"--explains why many statistically significant comparisons don't hold up. *American Scientist*, 102(6), 460-466.

Goldewijk, K.K., Beusen, A., Van Drecht, G., and De Vos, M. 2011. The HYDE 3.1 spatially explicit database of human‐induced global land‐use change over the past 12,000 years. *Global Ecology and Biogeography*, 20(1), 73-86.

Greenland, S., Senn, S.J., Rothman, K.J., Carlin, J.B., Poole, C., Goodman, S.N. and Altman, D.G. 2016. Statistical tests, P values, confidence intervals, and power: a guide to misinterpretations. *European Journal of Epidemiology*, 31(4), 337-350.

Greenland, S. 2019. Valid *P*-values behave exactly as they should: Some misleading criticisms of *P*-values and their resolution with *S*-values. *The American Statistician*, 73(sup1), 106-114.

Guyatt, G.H., Oxman, A.D., Vist, G., Kunz, R., Brozek, J., Alonso-Coello, P., Montori, V., Akl, E.A., Djulbegovic, B., Falck-Ytter, Y. and Norris, S.L. 2011a. GRADE guidelines: 4. Rating the quality of evidence—study limitations (risk of bias). *Journal of Clinical Epidemiology*, 64(4), pp.407-415.

Guyatt, G.H., Oxman, A.D., Kunz, R., Brozek, J., Alonso-Coello, P., Rind, D., Devereaux, P.J., Montori, V.M., Freyschuss, B., Vist, G. and Jaeschke, R. 2011b. GRADE guidelines 6. Rating the quality of evidence—imprecision. *Journal of Clinical Epidemiology*, 64(12), pp.1283-1293.

Hill, M.O. 2012. Local frequency as a key to interpreting species occurrence data when recording effort is not known. *Methods in Ecology and Evolution*, *3*(1), pp.195-205.

Hurlbert, S.H. and Lombardi, C.M. 2009. Final collapse of the Neyman-Pearson decision theoretic framework and rise of the neoFisherian. *Annales Zoologici Fennici*, 46(5), pp. 311-349.

Ioannidis, J.P.A. 2018. Meta-research: Why research on research matters. *PLoS Biology* 16(3): e2005468. https://doi.org/10.1371/journal.pbio.2005468

Jetz, W., McGeoch, M. A., Guralnick, R., Ferrier, S., Beck, J., Costello, M. J., ... and Turak, E. 2019. Essential biodiversity variables for mapping and monitoring species populations. *Nature Ecology & Evolution*, 3(4), 539-551.

Kéry, M. & Royle, J.A. 2016. *Applied Hierarchical Modelling in Ecology: Analysis of distribution, abundance and species richness in R and BUGS. Vol. 1.* Academic Press, London, UK.

Lortie, C. J., Stewart, G., Rothstein, H. and Lau, J. 2015. How to critically read ecological meta-analyses. *Research Synthesis Methods*, 6(2), 124–133. doi: 10.1002/jrsm.1109

Nøttestad, L., Krafft, B.A., Anthonypillai, V., Bernasconi, M., Langård, L., Mørk, H.L. and Fernö, A., 2015. Recent changes in distribution and relative abundance of cetaceans in the Norwegian Sea and their relationship with potential prey. *Frontiers in Ecology and Evolution*, 2, p.83.

Page M.J., Moher, D., Bossuyt, P.M., Boutron, I., Hoffmann, T.C., Mulrow, C.D. et al. 2021. PRISMA 2020 explanation and elaboration: updated guidance and exemplars for reporting systematic reviews. *BMJ*, 372:n160 doi:10.1136/bmj.n160

Pescott, O.L., Powney, G.D. and Roy, D.B. 2016 *Approaches to Bayesian occupancy modelling for habitat quality assessment.* Wallingford, NERC/Centre for Ecology & Hydrology, 23pp. doi:[10.13140/RG.2.2.15726.25927](https://doi.org/10.13140/RG.2.2.15726.25927)

Pescott, O.L., Humphrey, T.A., Stroh, P.A. and Walker, K.J. 2019a. Temporal changes in distributions and the species atlas: How can British and Irish plant data shoulder the inferential burden? *Br. Irish Bot.* 1, 250–282. doi:10.33928/bib.2019.01.250

Pescott, O. L., Walker, K. J., Harris, F., New, H., Cheffings, C. M., Newton, N., Jitlal, M., Redhead, J., Smart, S. M., & Roy, D. B. 2019b. The design, launch and assessment of a new volunteer-based plant monitoring scheme for the United Kingdom. *PLoS One*, 14, 1–30. https://doi.org/10.1371/journal.pone.0215891

Peters, R.H. 1991. *A Critique for Ecology*. Cambridge, UK: Cambridge University Press.

Phillips, S. J., Dudík, M., Elith, J., Graham, C. H., Lehmann, A., Leathwick, J., & Ferrier, S. 2009. Sample selection bias and presence-only distribution models: Implications for background and pseudo-absence data. *Ecological Applications*, 19, 181–197. https://doi.org/10.1890/ 07-2153.1

Royle, J.A. 2006. Site occupancy model with heterogeneous detection probabilities. *Biometrics*, 60, 97-102.

Shirey, V., Belitz, M.W., Barve, V. and Guralnick, R., 2021. A complete inventory of North American butterfly occurrence data: narrowing data gaps, but increasing bias. *Ecography*, 44(4), 537-547.

Shmueli, G. 2010. To explain or to predict? *Statistical Science*, 25(3), 289-310.

Speed, J. D. M., Bendiksby, M., Finstad, A. G., Hassel, K., Kolstad, A. L., & Prestø, T. 2018. Contrasting spatial, temporal and environmental patterns in observation and specimen based species occurrence data. *PLoS ONE*, 13, 1–17. https://doi.org/10.1371/journ al.pone.0196417

Sterne, J.A., Hernán, M.A., Reeves, B.C., Savović, J., Berkman, N.D., Viswanathan, M., Henry, D., Altman, D.G., Ansari, M.T., Boutron, I. and Carpenter, J.R. 2016. ROBINS-I: a tool for assessing risk of bias in non-randomised studies of interventions. *BMJ*, 355: i4919 doi:10.1136/bmj.i4919.

van Strien, A.J., van Duuren, L., Foppen, R.P.B. and Soldaat, L.L., 2009. A typology of indicators of biodiversity change as a tool to make better indicators. *Ecological Indicators*, 9(6), 1041-1048.

Woodcock, B.A., Isaac, N.J., Bullock, J.M., Roy, D.B., Garthwaite, D.G., Crowe, A. and Pywell, R.F. 2016. Impacts of neonicotinoid use on long-term population changes in wild bees in England. *Nature Communications*, 7(1), 1-8.

1. More generally, the term “event” is defined in the Darwin Core ontology, used by GBIF and other databases, as “[a]n action that occurs at some location during some time” (https://dwc.tdwg.org/terms/). [↑](#footnote-ref-1)
2. For example, compare the development and use of site-occupancy models for data specifically relating to visits to sites (Kéry & Royle, 2016), to the “frequency scaling using local occupancy” (FreScaLO) technique developed by Hill (2012) for coarser spatio-temporal data, such as those often found in species distribution atlases, and/or summarised at such coarser scales. [↑](#footnote-ref-2)
3. Whilst this is not the place for diversions into the appropriateness of Neyman-Pearson, or other types, of hypothesis testing frameworks in this area of ecology, we recommend that readers should be familiar with the logic behind the various different uses (and mis-uses) of *P*-values, rather than merely assuming that “significance testing” actually makes sense for their research question (e.g. see Greenland et al., 2016; Greenland, 2019; Hurlbert & Lombardi, 2009). [↑](#footnote-ref-3)
4. Note that this could equally be seen as an issue relating to taxonomic extent or coverage rather than resolution. In the taxonomic domain we take resolution to mean taxonomic level (i.e. family, genus, species etc.), whilst extent would refer to the extent of “horizontal” coverage across a phylogeny. [↑](#footnote-ref-4)
5. Although we note that the temporal distribution of occurrence data may still be a legitimate concern even for purely spatial models if stationarity assumptions about environmental driver/response relationships are implausible over long time periods. [↑](#footnote-ref-5)
6. For example, Kéry & Royle (2016, p. 559) provide the following example: “if multiple surveys [i.e. replicates within a temporal closure period] are only undertaken at the “better” sites, where density and therefore detection probability (*p*) may be higher on average, the resulting estimate of *p* will be biased high with respect to all sites and therefore the occupancy estimator will be biased low”. In addition, “unmodelled site-specific heterogeneity in detection will lead to underestimates of occupancy” (Kéry & Royle, 2016, pp. 560- 561). Other potential problems with biases specific to site-occupancy models also exist (e.g. see Pescott et al., 2016; Pescott et al., 2019a). [↑](#footnote-ref-6)
