# Supplementary material for: ROBITT: A tool for assessing the risk‐of‐bias in studies of temporal trends in ecology
Source: Methods Ecol Evol. 2022 Apr 6;13(7):1497–507. doi: 10.1111/2041-210X.13857 (PMC9541136; doi:10.1111/2041-210X.13857)
Supplement: Supplementary file 3 — Appendix S3 Supplementary Material 3 [file MEE3-13-1497-s005.docx]

**Tool development**

A prototype version of the ROBITT tool was created by RB, GP, and OP (authors). In a similar way to other RoB tools (e.g. Sterne et al., 2016), this prototype was refined over the course of two online workshops in which participants (the other authors) gave critical feedback. During the first workshop, participants were asked to review the tool, guided, but not restricted, by a broad set of prompting questions (supplementary material **3**). During the second workshop, each participant presented their feedback. Feedback was grouped into broad themes that warranted discussion in terms of establishing a consensus across participants on the content and presentation of the tool. The workshop chair (OP) outlined the perceived consensus after open discussion on each point, and participants were asked for further comments. The ROBITT tool and guidance document were then updated and circulated to participants for additional feedback (this process used an online word processing tool, so that all feedback was visible to all participants). The tool and guidance versions presented with this paper are the final result of this consensus-forming process.

**Prompting questions for the ROBITT tool consensus workshops**

Note that participants were given these to assist with structuring their feedback and thinking, but there was ultimately no requirement to address all of these, nor were other topics disallowed.

1. Can you imagine how this [tool] could be applied to questions in your area of research on species occurrence data? (Thinking through how you would actually use the [ROBITT] tool will likely result in the most practical suggestions here.)
2. Are there any concepts that are not well explained to you?
3. Are there missing inferential domains that you think should be dealt with separately, or noted as comments?
4. Do you think it would be useful/possible to deal with other general types of biases within this tool (e.g. model mis-specification, cognitive [biases], data dredging/forking paths etc.)?
5. Do you think it could be simplified/streamlined, or, conversely, aspects that could be more detailed?
6. Are you happy with the format (simple set of questions to answer in Word without prespecified formatting such as a table)? Could we improve it (e.g. would an RShiny app be more useful)?
7. How is supporting/explanatory information best communicated (as currently^[[1]](#footnote-1)^/separate document/endnotes/the [research] paper itself/other)?
8. How do we increase uptake in the research community? (Might be suggestions for tool/paper, or other approaches).
9. Any other comments or points that you wish to make.

**Reference**

Sterne, J.A., Hernán, M.A., Reeves, B.C., Savović, J., Berkman, N.D., Viswanathan, M., Henry, D., Altman, D.G., Ansari, M.T., Boutron, I., Carpenter, J.R., Chan, A.W., Churchill, R., Deeks, J.J., Hróbjartsson, A., Kirkham, J., Jüni, P., Loke, Y.K., Pigott, T.D., Ramsay, C.R., et al., 2016. ROBINS-I: A tool for assessing risk of bias in non-randomised studies of interventions. *BMJ* 355, 4–10. https://doi.org/10.1136/bmj.i4919

1. At the time of the workshop, guidance was only in the form of brief notes presented within the tool itself. The separate guidance document format was one result of the workshop. [↑](#footnote-ref-1)
