# Supplementary material for: ROBITT: A tool for assessing the risk‐of‐bias in studies of temporal trends in ecology
Source: Methods Ecol Evol. 2022 Apr 6;13(7):1497–507. doi: 10.1111/2041-210X.13857 (PMC9541136; doi:10.1111/2041-210X.13857)
Supplement: Supplementary file 4 — Appendix S4 Supplementary Material 4 [file MEE3-13-1497-s001.docx]

[This page can be deleted.]

# ROBITT (“Risk Of Bias In studies of Temporal Trends in ecology”) tool

Please ensure that you understand the premise of this document as outlined in the following paper and the associated guidance document:

Boyd, R.J., Powney, G.D., Burns, F., Danet, A., Duchenne, F., Grainger, M., Jarvis, S.G., Martin, G., Nilsen, E.B., Porcher, E., Stewart, G.B., Wilson, O.J., Pescott, O.L. In prep. ROBITT: a tool for assessing the risk of bias in studies of temporal trends in ecology. Prepared for submission to *Methods in Ecology and Evolution*

To complete this form, please delete all instances of <insert text> and replace with your own text and/or figures.

# Contents

[ROBITT (“Risk Of Bias In studies of Temporal Trends in ecology”) tool](#_gjdgxs) 1

[Contents](#_30j0zll) 1

[Iteration](#_1fob9te) 2

Research statement and pre-bias assessments 2

[Statistical population of interest](#_3znysh7) 2

[Inferential goals](#_2et92p0) 2

[Data provenance](#_tyjcwt) 2

[Data processing](#_3dy6vkm) 2

[Bias assessment and mitigation](#_1t3h5sf) **2**

[Assessment resolution](#_4d34og8) 2

[Geographic domain](#_2s8eyo1) 3

[Environmental domain](#_17dp8vu) 3

[Taxonomic domain (or other organismal domain, e.g., phylogenetic, trait space etc.)](#_3rdcrjn) 3

[Other potential biases](#_26in1rg) 3

[Supporting references](#_35nkun2) 4

# Iteration

**1.1 ROBITT iteration number**

| **Iteration** | **Comments** |
| --- | --- |
| 1 | NA |

# Research statement and pre-bias assessments

## Statistical population of interest

**2.1 Define the statistical target population about which you intend to make inferences.**

| **Domain** | **Extent** | **Resolution** |
| --- | --- | --- |
| Geographic | South and Central America | 1⁰ grid cells |
| Temporal | 1950-2019 | Decadal |
| Taxonomic (or other relevant organismal domain such as functional group) | Hummingbirds (Trochilidae) | Species |
| Environmental | NA | NA |

## Inferential goals

**2.2 What are your inferential goals?**

I intend to estimate changes in hummingbird range sizes in South and Central America (SCA) over the period 1950 to 2019. Specifically, I want to estimate decadal changes range size at the 1° grid cell scale.

## Data provenance

**2.3 From where were your data acquired (please provide citations, including a DOI, wherever possible)? What are their key features in respect of the inferential aims of your study (see the guidance document for examples)?**

I downloaded all data on hummingbird occurrences (presence-only) collected in South and Central America between 1950 and 2019 using the continent filter “South America” which includes South America, MesoAmerica and the Caribbean (GBIF, 2021). These data are available through the following doi: doi.org/10.15468/dl.duugu9. Around 50% of the data derive from preserved specimens and around 50% from human observations; a small number of records also derive from material samples, machine observations and living specimens (see Fig. 1 for a temporal breakdown of record type). I did not manually inspect the data any further; instead, I used the CoordinateCleaner package in R (Zizka et al., 2019) to remove records that are likely to be erroneous on several grounds (see my answer to question 1.4 below).


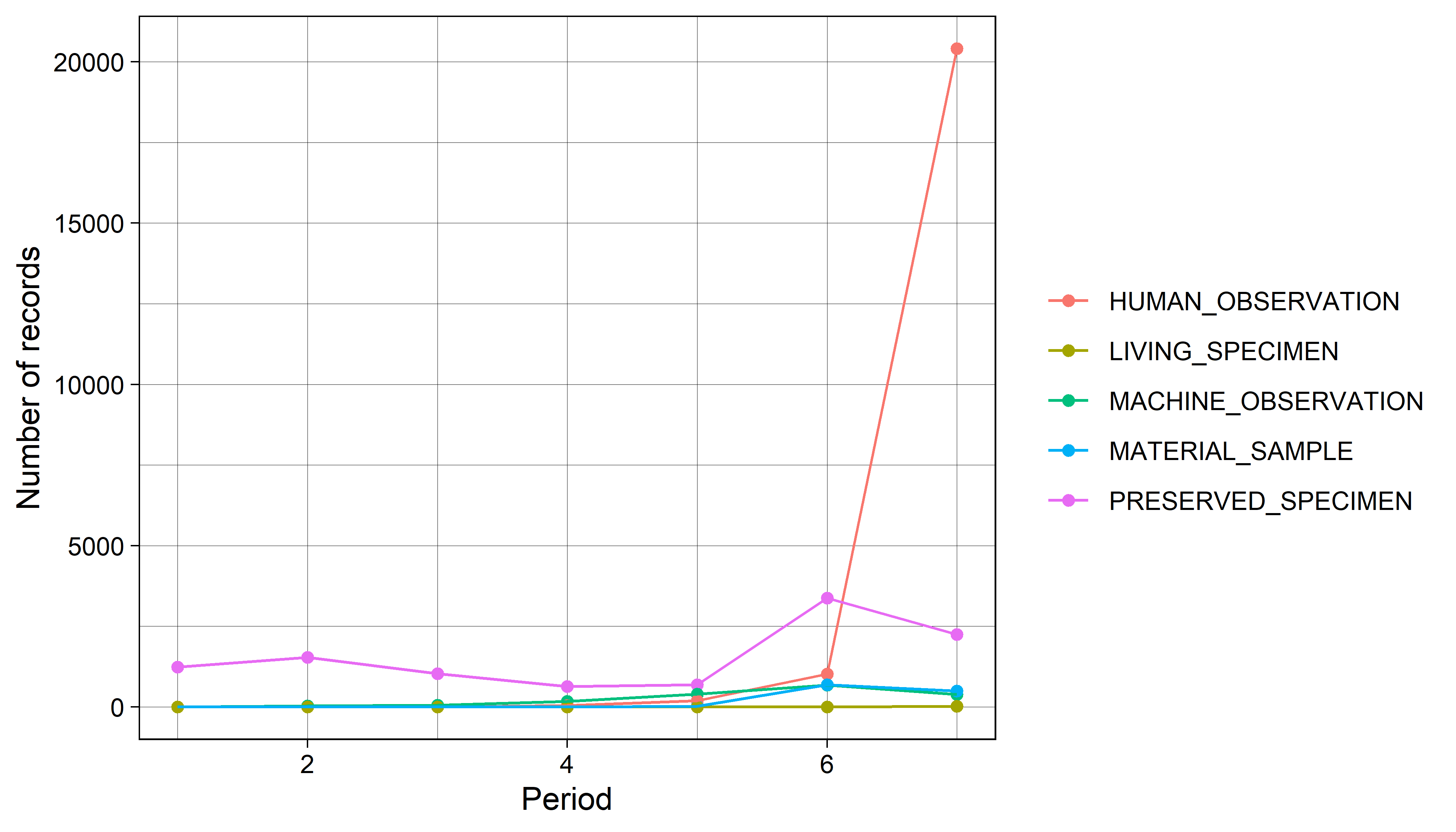


Figure 1. The number of records by record type in each decade.

## Data processing

**2.4 Provide details of, and the justification for, all of the steps that you have taken to clean the data described above prior to analyses.**

Having acquired the data as described above, I implemented two data cleaning steps. First, I removed all records that are not identified to species level (NA in the species field). Second, I used the clean_coordinates() function in the CoordinateCleaner package in R (Zizka et al., 2019) to remove records with probable spatial issues: coordinates matching country centroids, capital cities and biodiversity institutes; and coordinates with zero latitude or longitude, equal latitude and longitude, and in the ocean. The code used to clean the data can be found at <https://github.com/robboyd/ROBITT_example_code/blob/main/Humm_eg_clean_data>.

# Bias assessment and mitigation

## Assessment resolutions

**3.1 At what geographic, temporal and taxonomic resolutions (i.e. scales or grain sizes) will you conduct your bias assessment?**

I will conduct the bias assessment in 1⁰ grid cells and decadal time periods. Although I intend to draw inferences at the species level, it will not be possible to conduct the assessment at this resolution. This is because the data are presence-only so do not provide information on where and when a species was targeted unless it was found. Instead, I will assume that, when aggregated, the data for all species are representative of the distributions of sampling effort along the axes of space, time and taxonomy.

## Geographic domain

**3.2 Are the data sampled from a representative portion of geographical space in the domain of interest?**

To determine whether the data are randomly distributed across geographic space in SCA, I use the Nearest Neighbour Index [NNI; (Clark & Evans, 1954)]. The NNI is given as the ratio of the average of the nearest neighbor distances of the occurrence data to the average nearest neighbor distance of a simulated random distribution of the same density across SCA. Where the NNI < 1, the data are more clustered than a random distribution. We calculated the NNI for each decade (i.e. 1950-1959,.. 2010-2019; Fig. 2). The NNI is less than 0.4 in all cases; this indicates that the data are not randomly distributed across SCA.


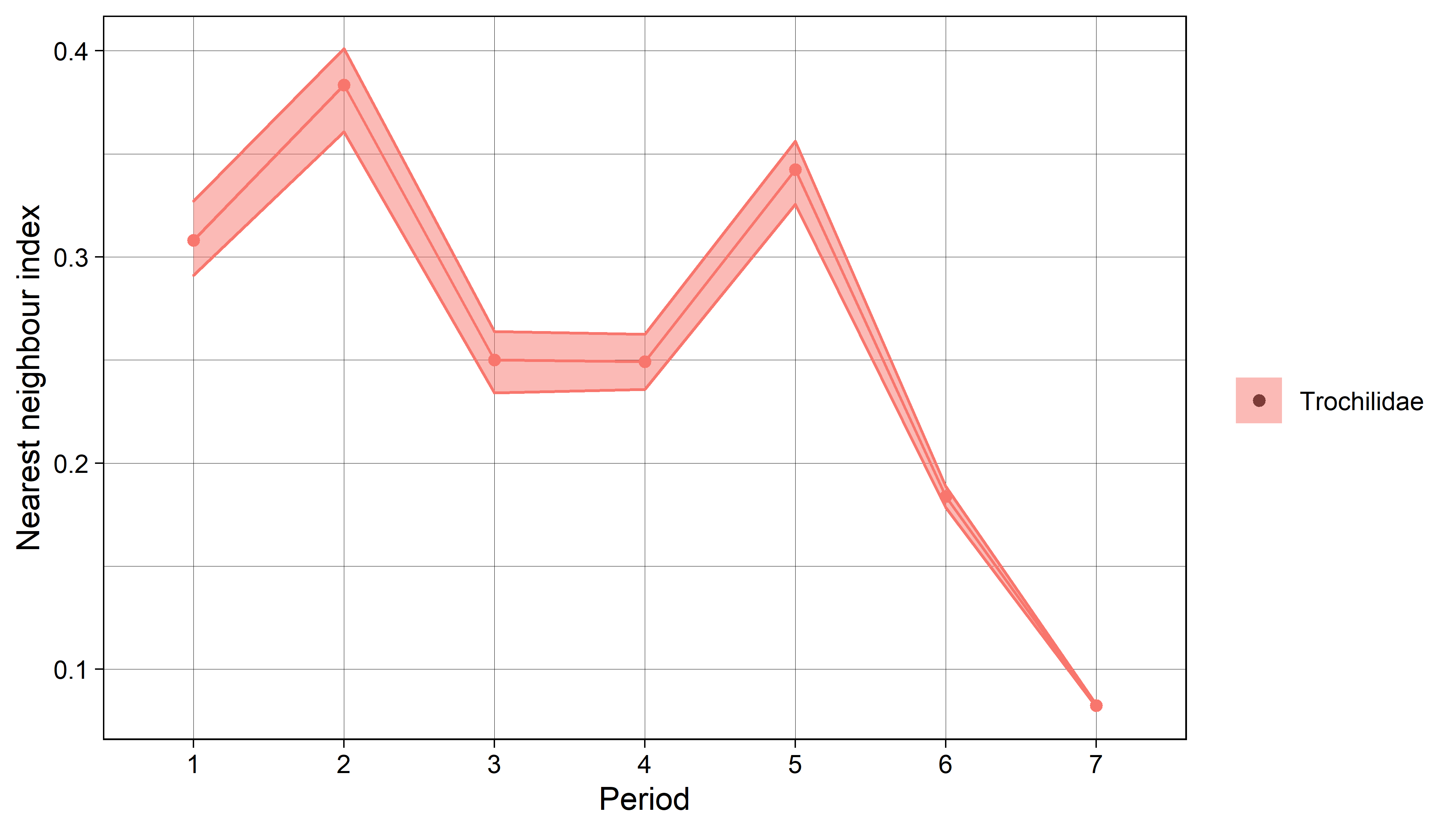


Figure 2. Nearest Neighbour Index (NNI) for the data in each decade. Values < 1 indicate that the data are more clustered than a random distribution. The shaded region delimits the 2.5^th^ and 97.5^th^ percentiles calculated by simulating 30 random distributions for each decade.

**3.3 Are your data sampled from the same portions of geographic space across time periods?**

To determine whether the data were sampled from the same portion of SCA over time, I map the number of decades in which each 1⁰ grid cell was sampled (had at least one record; Fig. 3). The majority of grid cells (of those which have been sampled) were sampled in three or fewer decades. There is, however, one relatively well-sampled portion of SCA: the Northwest region including Ecuador and Columbia. In these countries, many cells have been sampled in six or seven decades.


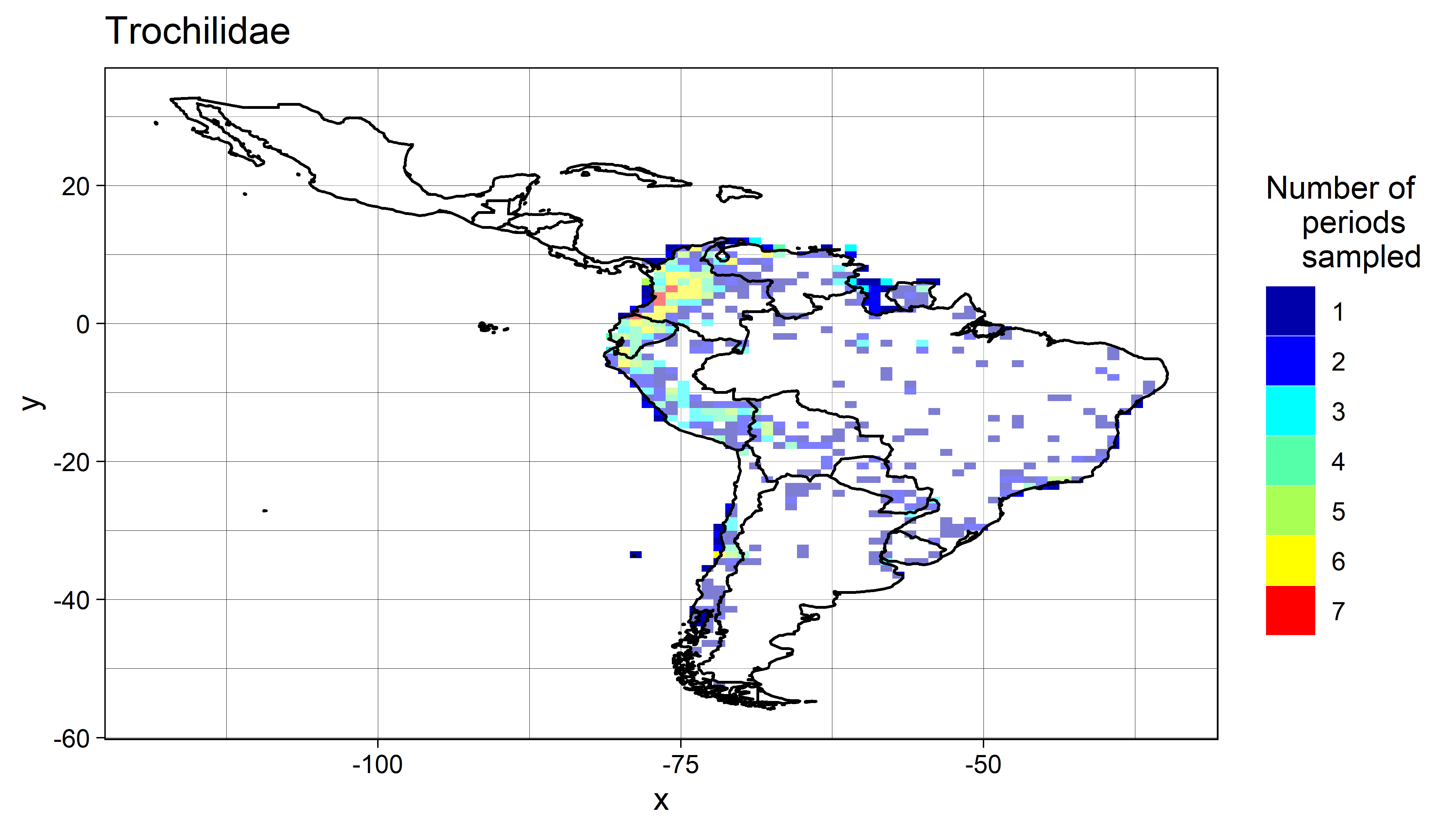


Figure 2. A map of SCA indicating the number of decades (1950-2019) in which each 1⁰ grid cell was sampled.

**3.4 If the answers to the above questions revealed any potential geographic biases, or temporal variation in geographic coverage, please explain, in detail, how you plan to mitigate them.**

Fig. 3 shows that different portions of SCA have been sampled over time. This will make it very difficult to obtain robust estimates of trends in species’ distributions at the continental scale. Instead, I will redefine the geographic domain of my target population from SCA to Ecuador and Columbia in the northwest of the continent – these countries are by far the most consistently sampled across decades (Fig. 3). I will not complete the remainder of this assessment; rather, I will begin a new iteration in which I focus solely on Ecuador and Colombia.

## Environmental domain

**3.5 Are your data sampled from a representative portion of environmental space in the domain of interest?**

<insert text>

**3.6 Are your data sampled from the same portion of environmental space across time periods?**

<insert text>

**3.7 If the answers to the above questions revealed any potential environmental biases, or temporal variation in environmental coverage, please explain, in detail, how you plan to mitigate them.**

<insert text>

## Taxonomic domain (or other organismal domain, e.g., phylogenetic, trait space etc.)

**3.8 Is the sampled portion of the taxonomic (or phylogenetic, trait or other space if more relevant) space representative of the taxonomic (or other) domain of interest?**

<insert text>

**3.9 Do your data pertain to the same taxa/taxonomic domain across time periods?**

<insert text>

**3.10 If the answers to the above questions revealed any potential taxonomic biases, or temporal variation in taxonomic coverage, please explain, in detail, how you plan to mitigate them.**

<insert text>

## Other potential biases

**3.11 Are there other potential temporal biases in your data that relate to variables other than ecological states?**

<insert text>

**3.12 Are you aware of any other potential biases not covered by the above questions that might cause problems for your inferences?**

<insert text>

**3.13 If questions 3.11 or 3.12 revealed any important potential biases, please explain how you will mitigate them.**

<insert text>

# Supporting references

Clark, P., & Evans, F. (1954). Distance to Nearest Neighbour as a Measure of Spatial Relationships in Populations. *Ecology*, *35*(4), 445–453. https://doi.org/10.1007/BF02315373

GBIF. (2021). *(28 April 2021) GBIF Occurrence Download: hummingbirds*. https://doi.org/https://doi.org/10.15468/dl.duugu9

Zizka, A., Silvestro, D., Andermann, T., Azevedo, J., Duarte Ritter, C., Edler, D., Farooq, H., Herdean, A., Ariza, M., Scharn, R., Svantesson, S., Wengström, N., Zizka, V., & Antonelli, A. (2019). CoordinateCleaner: Standardized cleaning of occurrence records from biological collection databases. *Methods in Ecology and Evolution*, *10*(5), 744–751. https://doi.org/10.1111/2041-210X.13152

[This page can be deleted.]

# ROBITT (“Risk Of Bias In studies of Temporal Trends in ecology”) tool

Please ensure that you understand the premise of this document as outlined in the following paper and the associated guidance document:

Boyd, R.J., Powney, G.D., Burns, F., Danet, A., Duchenne, F., Grainger, M., Jarvis, S.G., Martin, G., Nilsen, E.B., Porcher, E., Stewart, G.B., Wilson, O.J., Pescott, O.L. In prep. ROBITT: a tool for assessing the risk of bias in studies of temporal trends in ecology. Prepared for submission to *Methods in Ecology and Evolution*

To complete this form, please delete all instances of <insert text> and replace with your own text and/or figures.

# Contents

[ROBITT (“Risk Of Bias In studies of Temporal Trends in ecology”) tool](#_gjdgxs) 1

[Contents](#_30j0zll) 1

[Iteration](#_1fob9te) 2

Research statement and pre-bias assessments 2

[Statistical population of interest](#_3znysh7) 2

[Inferential goals](#_2et92p0) 2

[Data provenance](#_tyjcwt) 2

[Data processing](#_3dy6vkm) 2

[Bias assessment and mitigation](#_1t3h5sf) **2**

[Assessment resolution](#_4d34og8) 2

[Geographic domain](#_2s8eyo1) 3

[Environmental domain](#_17dp8vu) 3

[Taxonomic domain (or other organismal domain, e.g., phylogenetic, trait space etc.)](#_3rdcrjn) 3

[Other potential biases](#_26in1rg) 3

[Supporting references](#_35nkun2) 4

# Iteration

**1.1 ROBITT iteration number**

| **Iteration** | **Comments** |
| --- | --- |
| 2 | Initially, I intended to draw inferences about changes in hummingbird distributions across South and Central America. However, for most of the continent, there were very few grid cells that had been sampled consistently over time. By far the best-sampled portion of the continent is the sub-tropical Andean region in Ecuador and Colombia. Hence, I have redefined the geographic extent of my statistical population to focus exclusively on these countries in this iteration. |

# Research statement and pre-bias assessments

## Statistical population of interest

**2.1 Define the statistical target population about which you intend to make inferences.**

| **Domain** | **Extent** | **Resolution** |
| --- | --- | --- |
| Geographic | Ecuador and Columbia | 1⁰ grid cells |
| Temporal | 1950-2019 | Decadal |
| Taxonomic (or other relevant organismal domain such as functional group) | Hummingbirds (Trochilidae) | Species |
| Environmental | NA | NA |

## Inferential goals

**2.2 What are your inferential goals?**

I intend to estimate changes in hummingbird range sizes in Ecuador and Columbia (EC) over the period 1950 to 2019. Specifically, I want to estimate decadal changes in the species distributions measured at the 1° scale.

## Data provenance

**2.3 From where were your data acquired (please provide citations, including a DOI, wherever possible)? What are their key features in respect of the inferential aims of your study (see the guidance document for examples)?**

I downloaded all data on hummingbird occurrences (presence-only) collected in South and Central America between 1950 and 2019 using the continent filter “South America” which includes South America, MesoAmerica and the Caribbean (GBIF, 2021). These data are available through the following doi: doi.org/10.15468/dl.duugu9. I then used the countryCode field to remove any records collected outwith EC to reflect the new definition of my statistical population (as explained in my answer to question 1.1). The vast majority of records derive from human observations (20794) and preserved specimens (6023). Fig 1. shows the temporal breakdown of record type. I did not manually inspect the data any further; instead, I used the CoordinateCleaner package in R (Zizka et al., 2019) to remove records that are likely to be erroneous on several grounds (see my answer to question 2.4 below).


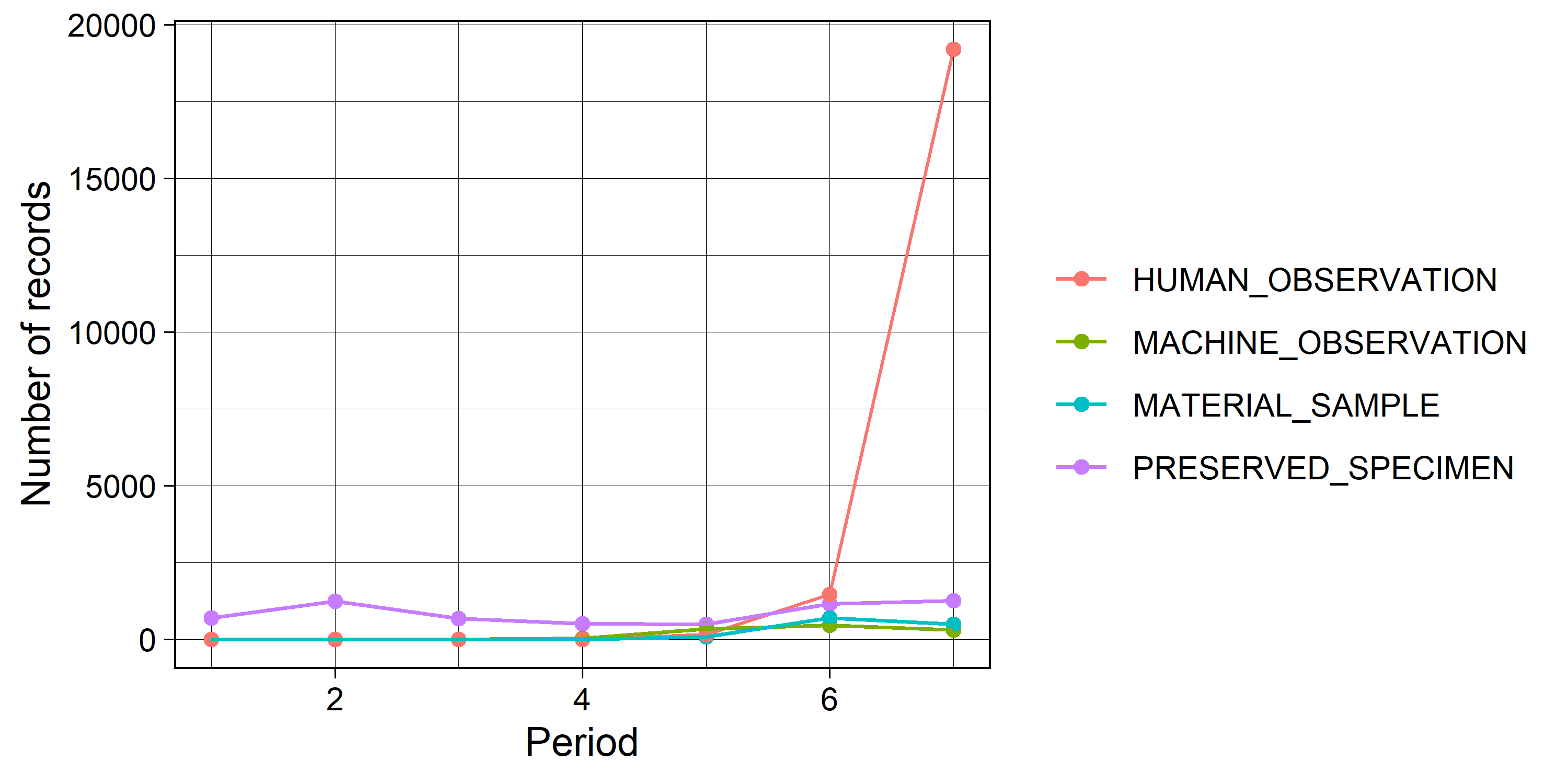


Figure 1. Basis of records per decade.

## Data processing

**2.4 Provide details of, and the justification for, all of the steps that you have taken to clean the data described above prior to analyses.**

Having acquired the data as described above, I implemented two data cleaning steps. First, I removed all records that are not identified to species level (NA in the species field). Second, I used the clean_coordinates() function in the CoordinateCleaner package in R (Zizka et al., 2019) to remove records with probable spatial issues: coordinates matching country centroids, capital cities and biodiversity institutes; and coordinates with zero latitude or longitude, equal latitude and longitude, and in the ocean. The code used to clean the data can be found at <https://github.com/robboyd/ROBITT_example_code/blob/main/Humm_eg_clean_data>.

# Bias assessment and mitigation

## Assessment resolutions

**3.1 At what geographic, temporal and taxonomic resolutions (i.e. scales or grain sizes) will you conduct your bias assessment?**

I will conduct the bias assessment in 1⁰ grid cells and decadal time periods. Although I intend to draw inferences at the species level, it will not be possible to conduct the assessment at this resolution. This is because the data are presence-only so do not provide information on where and when a species was targeted unless it was found. Instead, I will assume that, when aggregated, the data for all species are representative of the distributions of sampling effort along the axes of space, time and taxonomy.

The code used for this assessment can be found at <https://github.com/robboyd/ROBITT_example_code/blob/main/Humm_eg_assess_data>. Most of the analyses were conducted using the R package occAssess (Boyd et al., 2021).

## Geographic domain

**3.2 Are the data sampled from a representative portion of geographical space in the domain of interest?**

To determine whether the data are randomly distributed across geographic space in EC, I use the Nearest Neighbour Index [NNI; (Clark & Evans, 1954)]. The NNI is given as the ratio of the average of the nearest neighbor distances of the occurrence data to the average nearest neighbor distance of a simulated random distribution of the same density across EC. Where the NNI < 1, the data are more clustered than a random distribution. I calculated the NNI for each decade (i.e. 1950-1959,.. 2010-2019; Fig. 2). The NNI is less than 0.6 in all decades which indicates an appreciable departure from a random distribution. The departure is largest in decades six and seven.


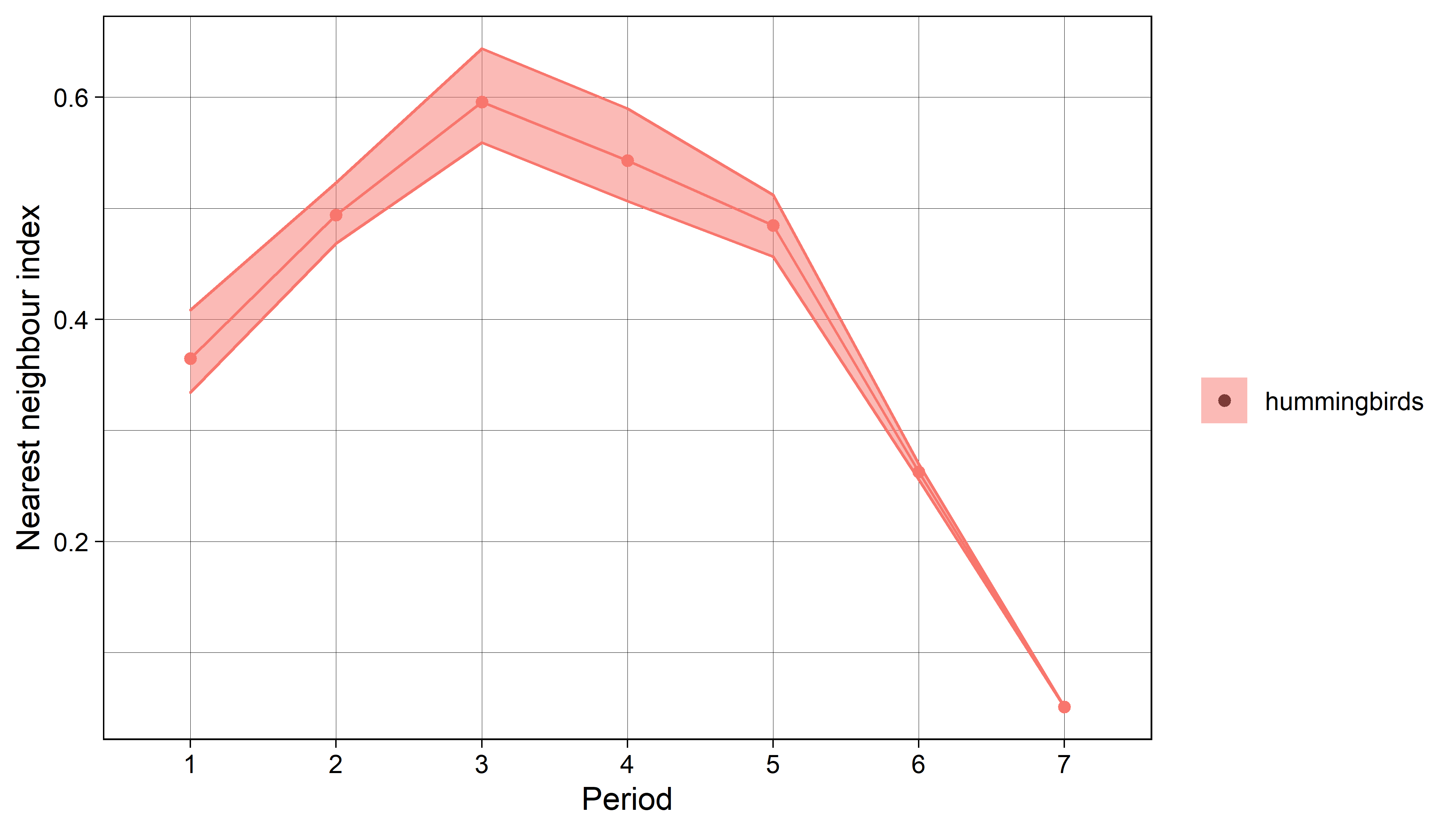


Figure 2. Nearest Neighbour Index (NNI) for the data in each decade. Values < 1 indicate that the data are more clustered than a random distribution. The shaded region delimits the 2.5^th^ and 97.5^th^ percentiles calculated by simulating 30 random distributions for each decade.

Whilst the NNI indicates that the data are not randomly distributed across EC, this do not necessarily indicate geographic sampling biases. In each decade, the records are concentrated in the subtropical Andean region (Fig. 3). Using a combination of GBIF data (from 1979-2017), expert range maps and expert knowledge of species’ elevation preferences, Ellis-Soto et al. (2021) fitted species distribution models for 276 species of hummingbird and showed that these upland areas are characterized by considerably higher species richness that the surrounding areas (see Fig. 5 of their paper). I suggest, therefore, that the distribution of records is likely representative of the distributions of the hummingbird species in the region.


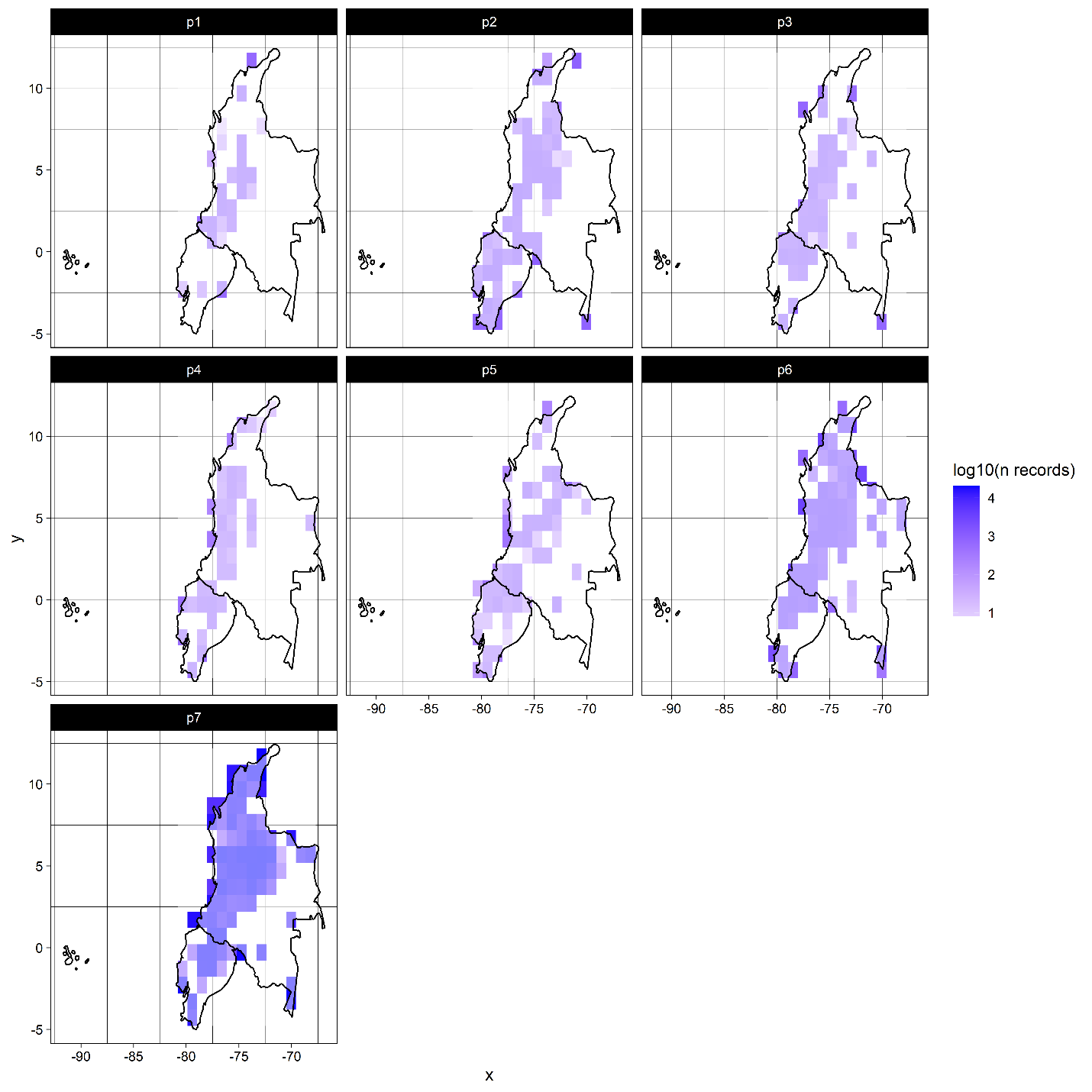


Figure 3. Density of records in 1⁰ grid cells across EC in each decade (p1.., p7).

**3.3 Are your data sampled from the same portions of geographic space across time periods?**

To determine whether the data were sampled from the same portion of SCA over time, I looked first to Fig. 3 which shows that roughly the same portion of EC has been sampled over time. More formally, I map the number of decades in which each 1⁰ grid cell was sampled (had at least one record) in Fig. 4. The majority of grid cells (of those which have been sampled) were sampled in three or more decades.


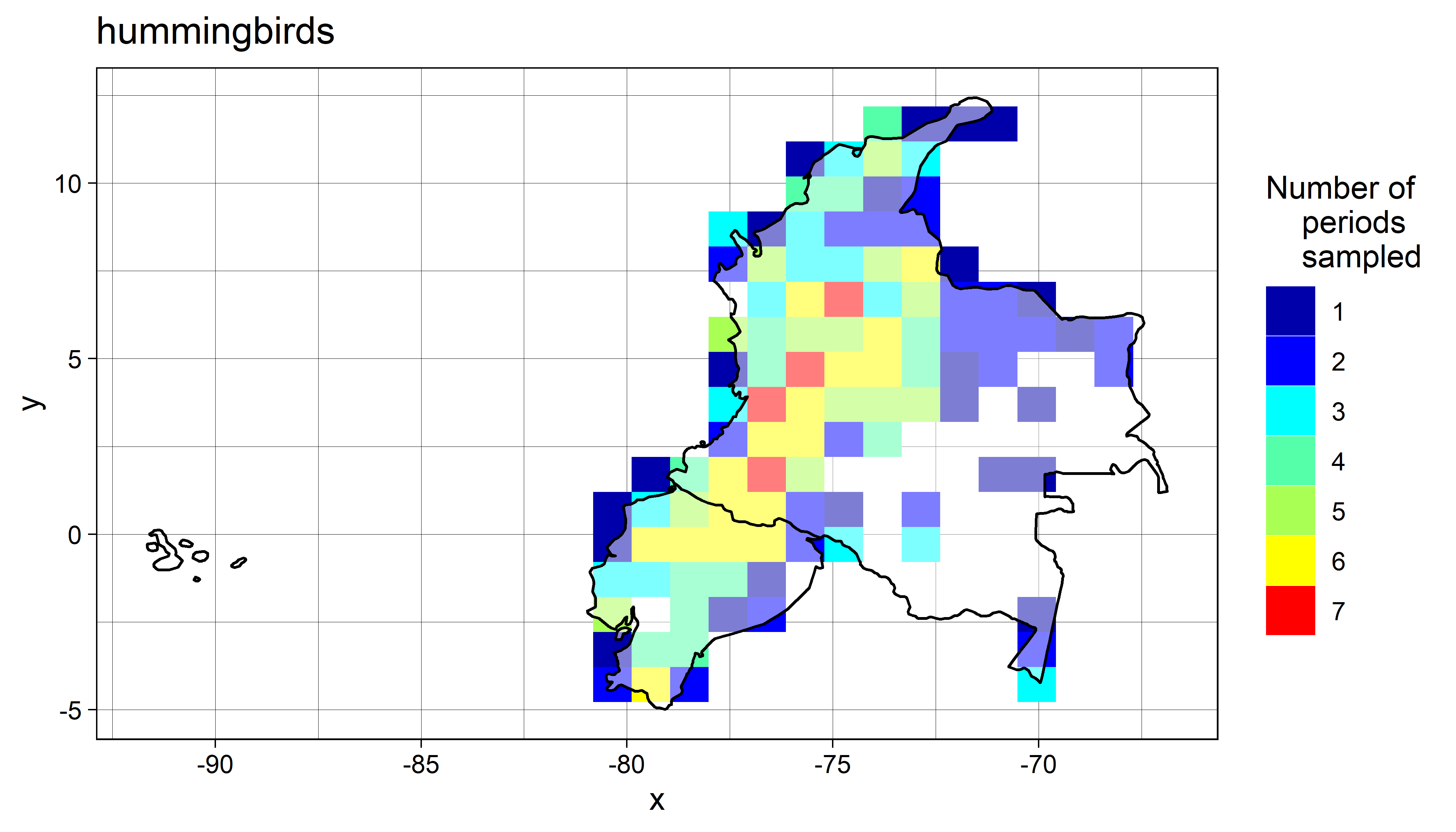


Figure 4. A map of EC indicating the number of decades (1950-2019) in which each 1⁰ grid cell was sampled.

**3.4 If the answers to the above questions revealed any potential geographic biases, or temporal variation in geographic coverage, please explain, in detail, how you plan to mitigate them.**

Whilst the geographic representativeness of the data is clearly much greater than at the continental scale (see previous iteration of this document), it is not possible to conclude that they are entirely free of bias. Hence, I will try to correct for these biases using two statistical models, each of which estimates temporal variation in species’ occupancy whilst attempting to correct for variation in recorder effort.

The first method is a variant of the “reporting rate” model (Franklin, 1999). This model predicts the probability of observing the focal species as function of time (decade in this case) and covariates related to sampling intensity and the focal species’ distribution. Previous studies have included list length – the number of species recorded in each cell per period – to control for spatio-temporal variation in recorder effort (e.g. Franklin 1999; Isaac et al., 2014). The rationale for including this covariate is that sampling intensity is likely to be greater where more species are recorded. It would not be appropriate to use the list length here for reasons discussed in my answer to question 3.12. Instead, I will use local inventory completeness, i.e. the proportion of species richness observed per cell per period, to control for recorder effort (see question 3.12 for details). Finally, I will include a random effect of grid cell (site; Roy et al. 2012). This allows for variation in reporting rates among sites.

The second method that I will use is that of Telfer et al. (2002). The first step in the Telfer method is to designate two time periods: I will treat the first and last three decades as periods one and two, respectively. The next step is to identify grid cells that have been sampled in both time periods; all other grid cells are removed from the analysis. This is how the method deals with temporal variation in spatial coverage. Removing all grid cells that were not sampled in both periods also limits the spatial extent of the resultant predictions which must be communicated appropriately. The proportion of remaining grid cells that each species was recorded in is then calculated and logit transformed. The logit transformed proportions for period 2 are regressed on those for period 1 and each species’ deviation from the fitted regression, its residual, is used an index of change relative to the group.

## Environmental domain

**3.5 Are your data sampled from a representative portion of environmental space in the domain of interest?**

It should be noted from the outset that assessing the representativeness of the data in environmental space is challenging because most environmental data are not available, to my knowledge, for the entire time period of interest.

To conduct a crude assessment of the climatic representativeness of the data, I define climate space in the geographic domain of interest as the first two principal components of the 19 worldclim bioclimatic variables (Fick & Hijmans, 2017) extracted at 1000 random locations across EC. These data represent averages over the period 1970-2000. Hence, I am assuming that the relative geographic distribution of environmental conditions remains constant over time. In Fig. 5 I show the distribution of the data in this reduced environmental space relative to the “background”. The data are were sampled from a subset of climate space which reflects the fact that hummingbirds are most diverse in the upland regions (Ellis-Sotto et al., 2021).


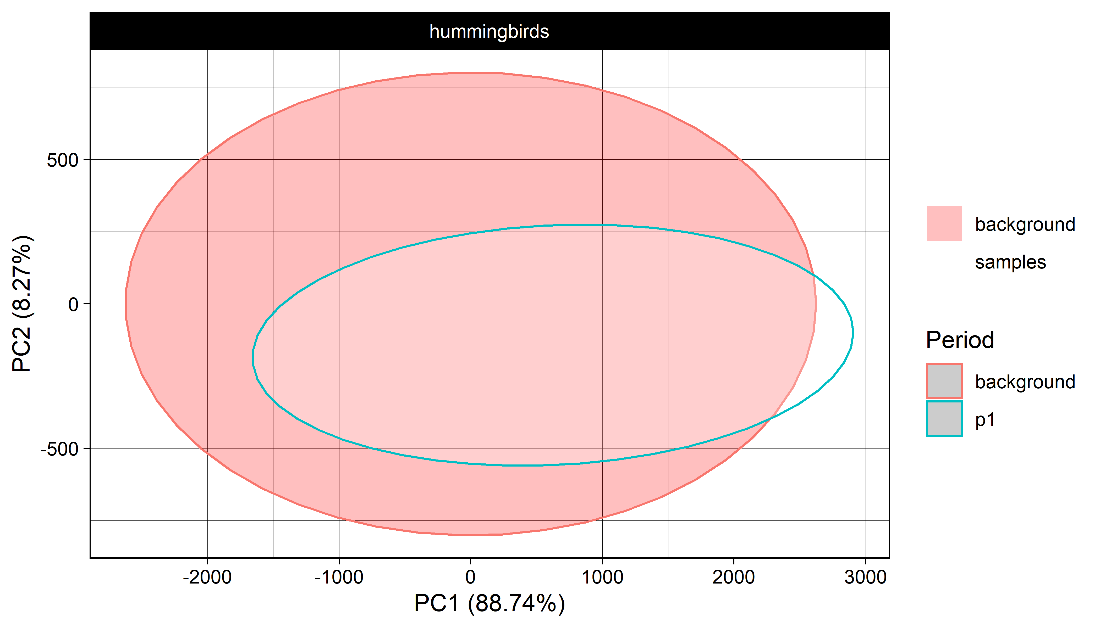


Figure 5. Distributions of the “background” sample (1000 randomly sampled points across EC) and the occurrence data in environmental space. Environmental space is defined as the first two principal components of the 19 worldclim bioclimatic variables extracted at the 1000 background points.

**3.6 Are your data sampled from the same portion of environmental space across time periods?**

To assess the degree to which the data are sampled from the same portion of environmental space over time, I switch my focus to elevation (altitude) because, to my knowledge, there are no temporally-varying climate data spanning 1950-2019. In addition to climate space, I also assessed whether the same altitudes were sampled each decade. Both hummingbird distributions and sampling intensity are strongly associated with altitude (Ellis-Sotto et al., 2021). I did not include altitude in my answer to question 3.5 because hummingbirds are known to occupy particular altitudinal ranges in EC which are not representative of the wider range of elevations.

In Fig. 6, I present the altitudinal distribution of the data in each decade using smoothed kernel density estimates (default parameters in the ggplot2 package; Wickham 2016). The distributions vary between decades; in particular, decade seven appears to differ from the rest.


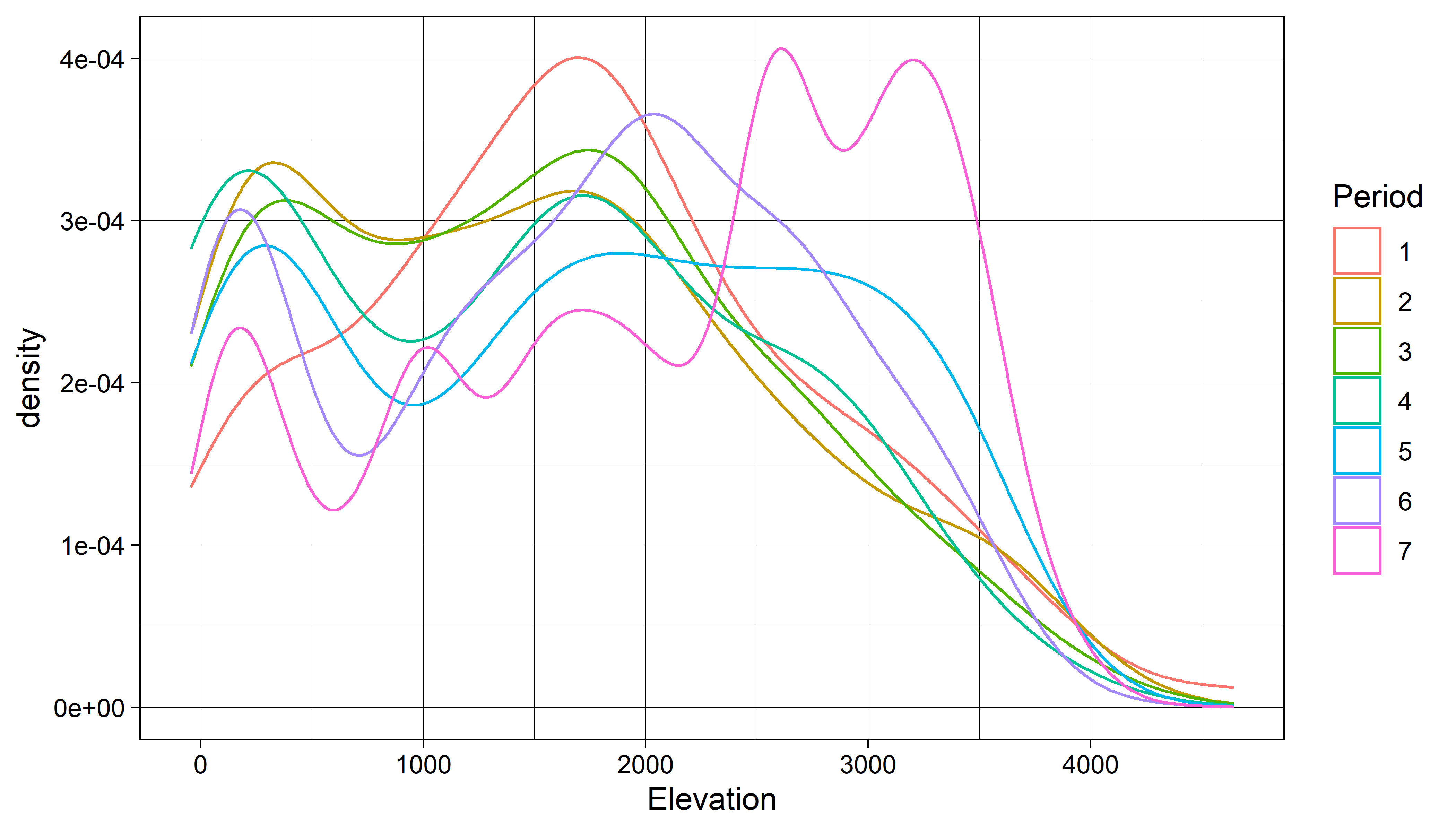


Figure 6. Smoothed kernel density estimates showing the altitudinal distribution of the data in each decade (meters).

**3.7 If the answers to the above questions revealed any potential environmental biases, or temporal variation in environmental coverage, please explain, in detail, how you plan to mitigate them.**

My answer to question 3.5 demonstrates that the data are not representative of climate space in EC. However, I do not consider this to be problematic for the inferential goal of estimating temporal trends in species’ distributions. The fact that the data are not representative of climate space across EC likely reflects the fact that they are sampled from the portions of geographic space in which hummingbird species richness is high (see my answer to questions 3.2 and 3.3).

Elevation is an important determinant of hummingbird occurrence (Ellis-Sotto et al., 2021) and Fig. 6 shows that different portions of altitudinal space have been sampled over time. To mitigate this sampling bias I will include a fixed effect of elevation range in the RR models described in my answer to question 3.4. The Telfer model is not suited to incorporation of covariates but only cells that have been sampled in periods one and two (decades 1-3 and 5-7, respectively) are included which will somewhat mitigate for varying elevation between periods.

## Taxonomic domain (or other organismal domain, e.g., phylogenetic, trait space etc.)

**3.8 Is the sampled portion of the taxonomic (or phylogenetic, trait or other space if more relevant) space representative of the taxonomic (or other) domain of interest?**

I have data for roughly half (173) of the approximately 340 species of hummingbird known to science (<http://home.olemiss.edu/~larryago/hummingbirds/species.html>). See Fig. 8 for a decadal breakdown of taxonomic representativeness.

Within the subset of species for which there are records, the data are not necessarily representative. Some taxa, such as rare and threatened species, tend be recorded relatively more often than others. To assess this “rarity bias”, I calculated each species’ time-varying prevalence as the number of 1° grid cells in which it has been recorded in each decade. Then, for each decade, I regressed the number of records for each species on their prevalence. I use the r^2^ values from each decadal regression as index of proportionality between species’ prevalence and the number of times they have been recorded (Fig. 7).

Fig. 7 indicates that, in most decades, rare species are recorded relatively more often than common species (values farther from one indicate greater biases). These biases are strongest in decades one, three and four.


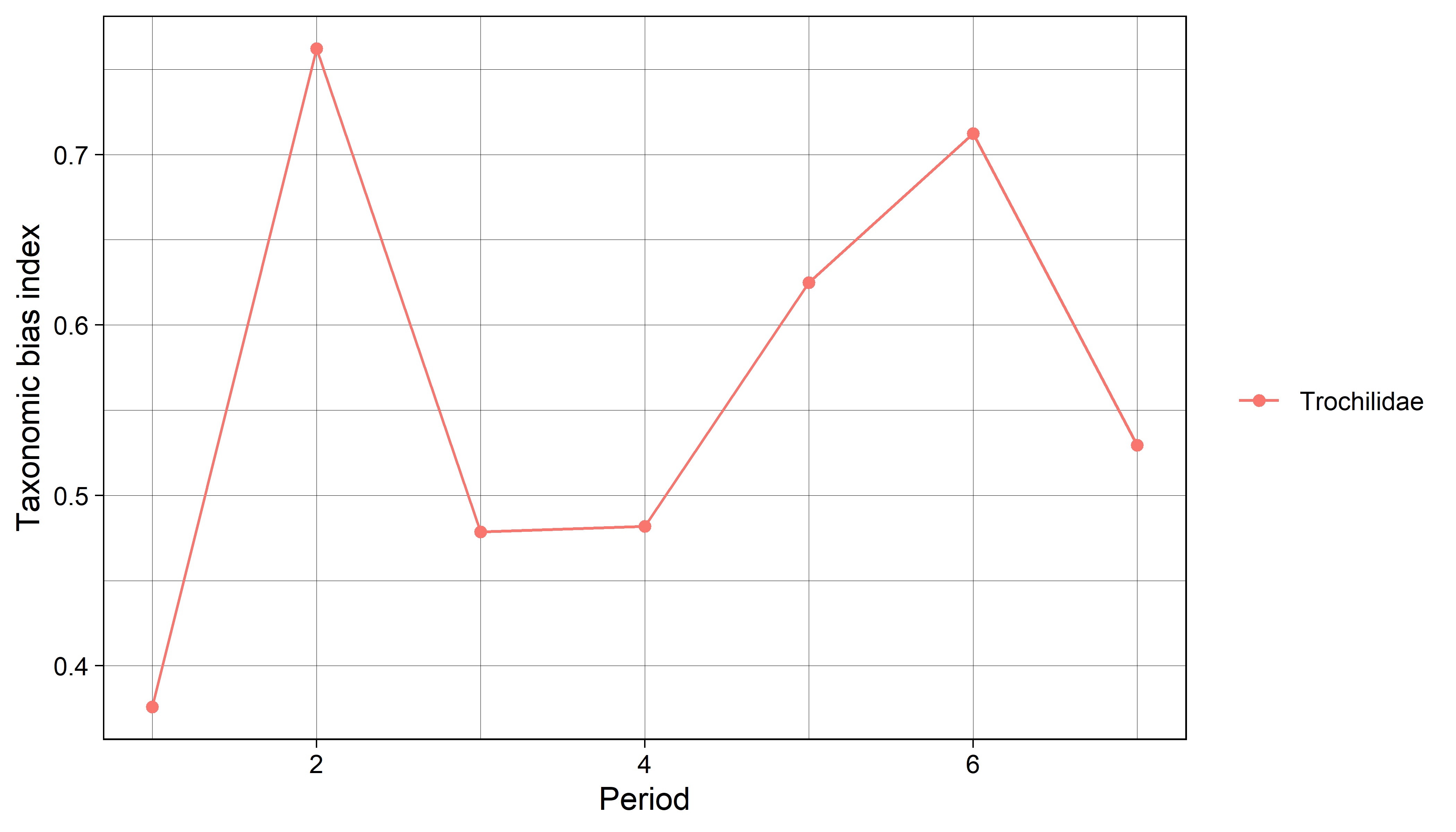


Figure 7. Taxonomic bias index indicating the extent to which rare species are over-sampled relative to commoner ones. Values further from one indicate a greater bias. The index is equal to the r^2^ from a regression of the number of records for each species on the number of grid cells in which they were recorded.

**3.9 Do your data pertain to the same taxa/taxonomic domain across time periods?**

To assess whether the same taxa are represented in the data across time periods, I first plot the number of species in each decade in Fig. 8. From this figure it is apparent that the number of species recorded in any one decade is lower than the total number of species in the dataset (173). This indicates inter-decadal variation in terms of which species are represented in the data. To investigate this further, I also performed a chi-squared test of independence on a contingency table in which the rows denote species and the columns denote time periods. This test indicates a significant difference in the composition of species in the data between decades (X^2^ = 10648 on 786 degrees of freedom, p = 0). In summary, there is strong inter-decadal variation in terms of which species have been sampled.


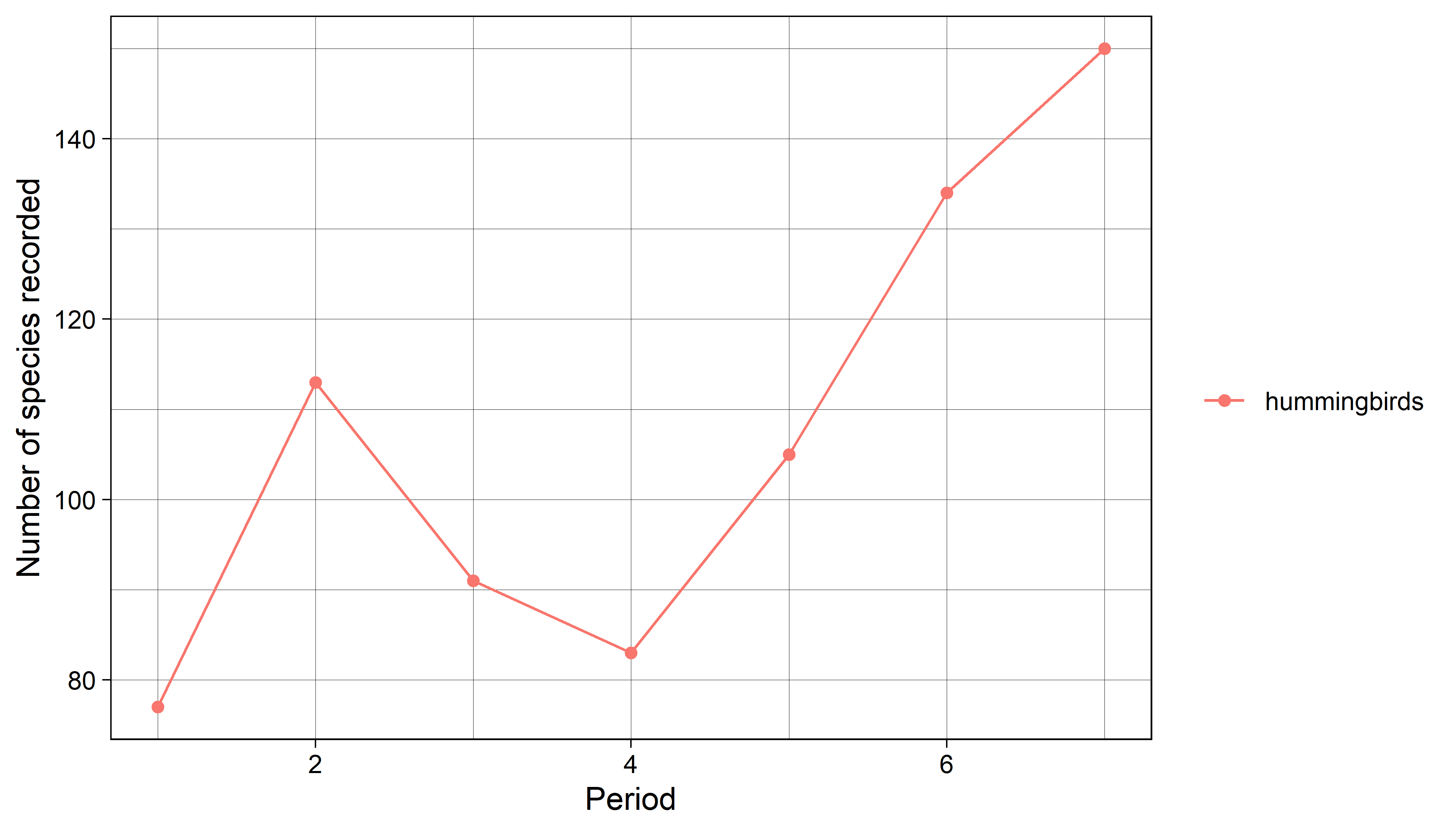


Figure 8. Sum of the number of species recorded in each decade.

**3.10 If the answers to the above questions revealed any potential taxonomic biases, or temporal variation in taxonomic coverage, please explain, in detail, how you plan to mitigate them.**

In my answer to question 3.4, I explained that I will use two statistical models to try and estimate temporal trends in species’ distributions whilst correcting for variation in sampling intensity. In light of the taxonomic biases revealed in this subsection, I will restrict the taxonomic extent of my statistical population to include only those species which were sampled in every decade, and make this clear when presenting my analysis. Forty species meet this criterion.

## Other potential biases

**3.11 Are there other potential temporal biases in your data that relate to variables other than ecological states?**

<insert text>

**3.12 Are you aware of any other potential biases not covered by the above questions that might cause problems for your inferences?**

In my answer to question 3.4 I stated that I will use the “reporting rate” model to estimate distributional changes and include a fixed effect of list length to account for variation in recorder effort. However, Ellis-Sotto et al. (2021) show that there is considerable spatial variation in species richness in EC. Hence, variation in list length might simply reflect variation in species richness not recorder effort.

**3.13 If questions 3.11 or 3.12 revealed any important potential biases, please explain how you will mitigate them.**

In my answer to question 3.12, I explained that list length could be confounded with local species richness. For this reason, I will substitute list length (number of species recorded) with the proportion of species recorded – i.e. inventory completeness per grid cell per decade – based on the data from Ellis-Sotto et al. (2021) and the inventories available at <https://mol.org/datasets/769f3b99-214e-4056-8c39-1200a6855943>.

# Supporting references

Boyd, R. J., Powney, G., Carvell, C., & Pescott, O. L. (2021). occAssess: An R package for assessing potential biases in species occurrence data. *Ecology and Evolution*, *August*. https://doi.org/10.1002/ece3.8299

Clark, P., & Evans, F. (1954). Distance to Nearest Neighbour as a Measure of Spatial Relationships in Populations. *Ecology*, *35*(4), 445–453. https://doi.org/10.1007/BF02315373

Ellis-Soto, D., Merow, C., Amatulli, G., Parra, J. L., & Jetz, W. (2021). Continental-scale 1 km hummingbird diversity derived from fusing point records with lateral and elevational expert information. *Ecography*, *44*(4), 640–652. https://doi.org/10.1111/ecog.05119

Fick, S. E., & Hijmans, R. J. (2017). WorldClim 2 : new 1-km spatial resolution climate surfaces for global land areas. *International Journal of Climatology*. https://doi.org/10.1002/joc.5086

Franklin, D. C. (1999). Evidence of disarray amongst granivorous bird assemblages in the savannas of northern Australia, a region of sparse human settlement. *Biological Conservation*, *90*(1), 53–68. https://doi.org/10.1016/S0006-3207(99)00010-5

GBIF. (2021). *(28 April 2021) GBIF Occurrence Download: hummingbirds*. https://doi.org/https://doi.org/10.15468/dl.duugu9

Roy, H. E., Adriaens, T., Isaac, N. J. B., Kenis, M., Martin, G. S., Brown, P. M. J., Hautier, L., Frost, R., Roy, D. B., Comont, R., Zindel, R., Vlaenderen, J. Van, Lane, B., & Gifford, C. (2012). declines of native European ladybirds. *Diversity and Distributions*, *18*, 717–725.

Telfer, M. G., Preston, C. D., & Rothery, P. (2002). A general method for measuring relative change in range size from biological atlas data. *Biological Conservation*, *107*(1), 99–109. https://doi.org/10.1016/S0006-3207(02)00050-2

Wickham, H. (2016). *ggplot2: Elegant Graphics for Data Analysis.* Springer-Verlag.

Zizka, A., Silvestro, D., Andermann, T., Azevedo, J., Duarte Ritter, C., Edler, D., Farooq, H., Herdean, A., Ariza, M., Scharn, R., Svantesson, S., Wengström, N., Zizka, V., & Antonelli, A. (2019). CoordinateCleaner: Standardized cleaning of occurrence records from biological collection databases. *Methods in Ecology and Evolution*, *10*(5), 744–751. https://doi.org/10.1111/2041-210X.13152
