# Supplementary material for: ROBITT: A tool for assessing the risk‐of‐bias in studies of temporal trends in ecology
Source: Methods Ecol Evol. 2022 Apr 6;13(7):1497–507. doi: 10.1111/2041-210X.13857 (PMC9541136; doi:10.1111/2041-210X.13857)
Supplement: Supplementary file 5 — Table S1 Supplementary Material 5 [file MEE3-13-1497-s003.docx]

Table 1. Existing risk-of-bias (RoB) tools, and broader approaches to study quality containing RoB elements, used across disciplines, with information on enforcement and citation-based impact. V = version; citation numbers estimated by Google Scholar 1-Feb-2022 where not otherwise attributed.

| **Tool** | **Field** | **Study/data type** | **Details** | **Community promotion** | **Reference(s)** | **Citations** |
| --- | --- | --- | --- | --- | --- | --- |
| Cochrane RoB tool | Medicine | Randomized controlled trials of medical interventions | Used to qualitatively stratify meta-analyses according to RoB | Assessment of RoB is regarded as an essential component of a systematic review in this area | V1: Higgins et al. (2011)  V2: Sterne et al. (2019) | >40,000 (Sterne et al. 2019) |
| Constraints on Generality statement | Psychology | Any inferential study | Engenders clear definition of the statistical population of interest and assesses external validity. Used at the primary research stage | Requested by some journals as a part of good practice in open science (e.g. Lindsay, 2019) | Simons et al. (2017) | 510 |
| GRADE | Medicine | Medical recommendations | Not only an RoB tool but contains RoB component (which could be another approach, e.g. ROBINS-I). Used to rate quality of evidence and grade strength of recommendations | Recognised approach for use with systematic reviews, health technology assessments, and clinical practice guidelines addressing alternative management options. | Guyatt et al. (2011); Schünemann et al. (2013) | 5657 (for the introductory paper of Guyatt et al. 2011 only) |
| PRISMA | Cross-discipline | Systematic reviews and meta-analyses | Not only a RoB tool but contains RoB components at both the individual study and overall review level | Endorsed and recommended by many journals (e.g. see Stevens et al. (2014) | V1: Moher et al. (2009)  V2: Page et al. (2021) | >60,000 reports (Page et al. 2021) |
| PROBAST | Medicine | Predictive modelling studies of diagnoses and prognoses | Used at either the primary research or systematic review stage | Endorsed and recommended by journals | Wolff et al. (2019) | 480 |
| RoBANS | Medicine | Non-randomized studies of medical interventions | Used at the systematic review stage | Endorsed and recommended by journals | Kim et al. (2013) | 661 |
| ROBINS-E | Public health | Non-randomized studies of exposure | Akin to ROBINS-I, but for studies of effects of exposure. Used at the systematic review stage | Endorsed and recommended by some journals | Bero et al. (2018) | 92 |
| ROBINS-I | Medicine | Non-randomized studies of medical interventions | Compares data to that of a hypothetical randomized trial. Designed primarily for use at the systematic review stage | Endorsed and recommended by journals | Sterne et al. (2016) | 5155 |

**References**

Bero, L., Chartres, N., Diong, J., Fabbri, A., Ghersi, D., Lam, J., Lau, A., McDonald, S., Mintzes, B., Sutton, P., Turton, J. L., & Woodruff, T. J. (2018). The risk of bias in observational studies of exposures (ROBINS-E) tool: Concerns arising from application to observational studies of exposures. *Systematic Reviews*, *7*(1), 1–11. https://doi.org/10.1186/s13643-018-0915-2

Guyatt, G., Oxman, A. D., Akl, E. A., Kunz, R., Vist, G., Brozek, J., Norris, S., Falck-Ytter, Y., Glasziou, P., Debeer, H., Jaeschke, R., Rind, D., Meerpohl, J., Dahm, P., & Schünemann, H. J. (2011). GRADE guidelines: 1. Introduction - GRADE evidence profiles and summary of findings tables. *Journal of Clinical Epidemiology*, *64*(4), 383–394. https://doi.org/10.1016/j.jclinepi.2010.04.026

Higgins, J. P. T., Altman, D. G., Gøtzsche, P. C., Jüni, P., Moher, D., Oxman, A. D., Savović, J., Schulz, K. F., Weeks, L., & Sterne, J. A. C. (2011). The Cochrane Collaboration’s tool for assessing risk of bias in randomised trials. *BMJ (Online)*, *343*(7829), 1–9. https://doi.org/10.1136/bmj.d5928

Kim, S. Y., Park, J. E., Lee, Y. J., Seo, H. J., Sheen, S. S., Hahn, S., Jang, B. H., & Son, H. J. (2013). Testing a tool for assessing the risk of bias for nonrandomized studies showed moderate reliability and promising validity. *Journal of Clinical Epidemiology*, *66*(4), 408–414. https://doi.org/10.1016/j.jclinepi.2012.09.016

Lindsay, D. S. (2019). Swan Song Editorial. *Psychological Science*, *30*(12), 1669–1673. https://doi.org/10.1177/0956797619893653

Moher, D., Liberati, A., Tetzlaff, J., & Altman, D. G. (2009). Preferred reporting items for systematic reviews and meta-analyses: The PRISMA statement. *BMJ (Online)*, *339*(7716), 332–336. https://doi.org/10.1136/bmj.b2535

Page, M. J., McKenzie, J. E., Bossuyt, P. M., Boutron, I., Hoffmann, T. C., Mulrow, C. D., Shamseer, L., Tetzlaff, J. M., Akl, E. A., Brennan, S. E., Chou, R., Glanville, J., Grimshaw, J. M., Hróbjartsson, A., Lalu, M. M., Li, T., Loder, E. W., Mayo-Wilson, E., McDonald, S., … Moher, D. (2021). The PRISMA 2020 statement: An updated guideline for reporting systematic reviews. *The BMJ*, *372*. https://doi.org/10.1136/bmj.n71

Schünemann, H., Brożek, J., Guyatt, G., & Oxman, A. (2013). *GRADE handbook*. https://gdt.gradepro.org/app/handbook/handbook.html

Simons, D. J., Shoda, Y., & Lindsay, D. S. (2017). Constraints on Generality (COG): A Proposed Addition to All Empirical Papers. *Perspectives on Psychological Science*, *12*(6), 1123–1128. https://doi.org/10.1177/1745691617708630

Sterne, J. A. C., Savović, J., Page, M. J., Elbers, R. G., Blencowe, N. S., Boutron, I., Cates, C. J., Cheng, H. Y., Corbett, M. S., Eldridge, S. M., Emberson, J. R., Hernán, M. A., Hopewell, S., Hróbjartsson, A., Junqueira, D. R., Jüni, P., Kirkham, J. J., Lasserson, T., Li, T., … Higgins, J. P. T. (2019). RoB 2: A revised tool for assessing risk of bias in randomised trials. *The BMJ*, *366*, 1–8. https://doi.org/10.1136/bmj.l4898

Sterne, J. A., Hernán, M. A., Reeves, B. C., Savović, J., Berkman, N. D., Viswanathan, M., Henry, D., Altman, D. G., Ansari, M. T., Boutron, I., Carpenter, J. R., Chan, A. W., Churchill, R., Deeks, J. J., Hróbjartsson, A., Kirkham, J., Jüni, P., Loke, Y. K., Pigott, T. D., … Higgins, J. P. (2016). ROBINS-I: A tool for assessing risk of bias in non-randomised studies of interventions. *BMJ (Online)*, *355*, 4–10. https://doi.org/10.1136/bmj.i4919

Stevens, A., Shamseer, L., Weinstein, E., Yazdi, F., Turner, L., Thielman, J., Altman, D. G., Hirst, A., Hoey, J., Palepu, A., Schulz, K. F., & Moher, D. (2014). Relation of completeness of reporting of health research to journals’ endorsement of reporting guidelines: Systematic review. *BMJ (Online)*, *348*(June), 1–29. https://doi.org/10.1136/bmj.g3804

Wolff, R. F., Moons, K. G. M., Riley, R. D., Whiting, P. F., Westwood, M., Collins, G. S., Reitsma, J. B., Kleijnen, J., & Mallett, S. (2019). PROBAST: A tool to assess the risk of bias and applicability of prediction model studies. *Annals of Internal Medicine*, *170*(1), 51–58. https://doi.org/10.7326/M18-1376
